# Effects of internally directed cognition on smooth pursuit eye movements: A systematic examination of perceptual decoupling

**DOI:** 10.3758/s13414-023-02688-3

**Published:** 2023-03-15

**Authors:** Živa Korda, Sonja Walcher, Christof Körner, Mathias Benedek

**Affiliations:** Department of Psychology, University of Graz, Graz, Austria

**Keywords:** Smooth pursuit, Eye behavior, Perceptual decoupling, Internally directed cognition, Internal attention

## Abstract

Eye behavior differs between internally and externally directed cognition and thus is indicative of an internal versus external attention focus. Recent work implicated perceptual decoupling (i.e., eye behavior becoming less determined by the sensory environment) as one of the key mechanisms involved in these attention-related eye movement differences. However, it is not yet understood how perceptual decoupling depends on the characteristics of the internal task. Therefore, we systematically examined effects of varying internal task demands on smooth pursuit eye movements. Specifically, we evaluated effects of the internal workload (control vs. low vs. high) and of internal task (arithmetic vs. visuospatial). The results of multilevel modelling showed that effects of perceptual decoupling were stronger for higher workload, and more pronounced for the visuospatial modality. Effects also followed a characteristic time-course relative to internal operations. The findings provide further support of the perceptual decoupling mechanism by showing that it is sensitive to the degree of interference between external and internal information.

**Supplementary Information:**

The online version contains supplementary material available at 10.3758/s13414-023-02688-3.

## Introduction

Where is your mind at the moment? Are you fully focused on the article in front of you or are you distracted by thoughts about an upcoming event? Even if your attention is not fully devoted to reading this article, you are still at least partially aware of what is written. Humans possess the ability to divide their cognitive resources and switch the focus between external and internal attention (Dixon et al., 2014; Verschooren et al., 2019). During the day, our attention is often simultaneously captured by information from external and internal worlds, thus getting us engaged in both externally directed cognition (EDC; processing of sensory inputs) and internally directed cognition (IDC; processing of internal, sensory-independent mental representations) (Benedek, 2018; Dixon et al., 2014; Schooler et al., 2011; Smallwood & Schooler, 2006). However, sharing of attentional resources comes with a cost, such as reduced accuracy in the task that is not in the current focus of attention (Verschooren et al., 2019). One of the proposed mechanisms to protect ongoing internal processes from interference by sensory input is *perceptual decoupling* (PDec), which refers to the phenomenon that attention gets decoupled from the sensory environment during IDC (Benedek, 2018; Schooler et al., 2011; Smallwood & Schooler, 2006). PDec has been suggested to be responsible for eye behavior differences observed between EDC and IDC, i.e., eye behavior becoming more variable and less determined by sensory characteristics (Annerer-Walcher et al., 2018, 2021; Smallwood et al., 2011), yet it is not clear how these effects depend on the characteristics of the internal task. Therefore, the present study aimed to conduct a systematic investigation of PDec effects on eye behavior. Specifically, we examined how PDec depends on variations of internal task modality (arithmetic vs. visuospatial) and of internal workload (low vs. high). We studied PDec in the context of smooth pursuit eye movements (SPEM), which allows us to analyze deviations from expected eye behavior continuously across time.

### Internally (IDC) and externally directed cognition (EDC)

Attention is a fundamental cognitive process helping us navigate through a world full of sensory stimuli and focusing on relevant information. In the last 20 years, cognitive psychology increasingly acknowledged the importance of self-generated thought, which fostered research on IDC (Benedek, 2018; Christoff et al., 2016; Zabelina & Andrews-Hanna, 2016). Besides the internal/external distinction, attentional focus can further be divided into reflexive or voluntary, which can result in spontaneous or goal-directed IDC, for example mind wandering versus mental calculation (Dixon et al., 2014; Smallwood, 2011). Importantly, all forms of EDC and IDC draw from common cognitive resources (Dixon et al., 2014; Verschooren et al., 2019). These resources can be proportionally distributed across tasks, depending on the task demands. In some cases, a combination of EDC and IDC is even required, for example in reading (Smallwood, 2011). Nonetheless, simultaneous engagement in both EDC and IDC often results in interference between processes, seen as a decreased performance on one or both tasks, indicating the limited availability of cognitive resources (Benedek, 2018; Dixon et al., 2014; Smallwood, 2011; Verschooren et al., 2019). Furthermore, Verschooren et al. (2019) showed that switching between a working memory task (IDC) and a perceptual task (EDC) interferes with task performance to the same extent as switching between two working memory or two perceptual tasks. Altogether, this suggests that common attentional resources are divided between any parallel cognitive activities. As a consequence, changes in EDC performance may be indicative of the level of internal load.

### Eye behavior differences between IDC and EDC

Research in the past decade has shown that eye behavior consistently differs between IDC and EDC (Annerer-Walcher et al., 2018, 2021; Benedek et al., 2017; Ceh et al., 2020, 2021; Hollander & Huette, 2022; Reichle et al., 2010; Smallwood et al., 2011; Smilek et al., 2010; Unsworth & Robison, 2016; Uzzaman & Joordens, 2011; Walcher et al., 2017). For example, studies on mind wandering during external tasks have typically reported increased blink rates as well as longer blink duration (Hollander & Huette, 2022; Smilek et al., 2010), fewer and longer fixations unrelated to the external environment (Reichle et al., 2010; Uzzaman & Joordens, 2011), and smaller baseline pupil diameter (PD) as well as increased PD variability (Smallwood et al., 2011; Unsworth & Robison, 2016). Findings varied across studies, but Faber et al. (2020) showed that gaze patterns during mind wandering are more alike when external tasks had comparable visuospatial demands. Specifically, they investigated mind wandering during six visual tasks with different spatial allocation, visual and discourse processing demands (e.g., reading, watching a film). Tasks with similar spatial allocation and visual processing demands (but not discourse processing demands) resulted in a similar gaze pattern during mind wandering, showing that eye behavior during IDC depends on the task’s visuospatial demands (Faber et al., 2020).

Eye behavior differences during mind wandering could be due to shifts between external and internal attention, but also due to shifts from goal-directed to spontaneous forms of thinking. Therefore, further studies compared effects of external and internal attention within goal-directed cognitive activities (e.g., reading vs. mental arithmetic). These works confirmed that various eye parameters are sensitive to the direction of attention (internal vs. external) even during goal-directed IDC, but specific changes again depended substantially on the type of external and internal task (Annerer-Walcher et al., 2018, 2021; Ceh et al., 2020, 2021; Gouraud et al., 2021; Walcher et al., 2017); only a few eye parameters appeared to be robustly associated with IDC independent of task type, including increased blink rate, pupil diameter variance, and fixation disparity variance (Annerer-Walcher et al., 2021). These findings indicate that eye behavior can serve as an index of external versus internal attention.

### Perceptual decoupling

What drives eye behavior differences between externally and internally directed cognition? Smallwood and Schooler (2006) argued that during mind wandering one becomes disengaged from the primary external task and enters a state where information processing is decoupled from the sensory environment, a mechanism referred to as *PDec* (Schooler et al., 2011; Smallwood & Schooler, 2015). As cognitive resources are limited, PDec is thought to shield ongoing internal processes from interference by external stimulation (Benedek et al., 2017; Smallwood, 2013). Consequently, PDec is not limited to mind wandering but also applies to goal-directed forms of IDC such as creative cognition involving imagination (Benedek, 2018). Furthermore, due to attentional resources being shared across cognitive domains, PDec from external visual information can even be observed when attention is focused on a different sensory modality (e.g., auditory vs. visual EDC; Hidaka & Ide, 2015; Malpica et al., 2020).

PDec is evidenced by eye behavior becoming less determined by the characteristics and dynamics of the visual environment. Relevant eye behaviors include gaze aversion (Doherty-Sneddon & Phelps, 2005; Mastroberardino & Vredeveldt, 2014; Vredeveldt et al., 2011), increased fixation disparity (i.e., staring into space; Annerer-Walcher et al., 2018), and increased blink rates and durations (Annerer-Walcher et al., 2018; Walcher et al., 2017), which all contribute to reduce the amount of visual information (Denkova et al., 2018; Faber et al., 2020; Walcher et al., 2017). Moreover, PDec was related to increased variability of eye behavior (Annerer-Walcher et al., 2021; Smallwood et al., 2011). Smallwood et al. (2011) showed spontaneous activity of PD during mind wandering, which was unrelated to the external task, thus indicating that the eyes were less constrained by the primary target and potentially coupled to imagined objects or scenes (i.e., internal coupling). Furthermore, Annerer-Walcher et al. (2021) extended these findings in the domain of goal-oriented IDC, showing that PD variation and fixation disparity variation consistently increase in the internal versus external tasks in three modality domains (i.e., arithmetic, verbal, visuospatial). However, PDec was not always accompanied by a change in eye behavior, but sometimes just by reduced processing of visual information (Annerer-Walcher et al., 2018; Malpica et al., 2020). For example, although participants perceived multiplication trials with distractors as more challenging, there was no effect on eye behavior (Annerer-Walcher et al., 2018), thus reinforcing the view that PDec is an attentional and not merely oculomotor phenomenon.

Besides eye behavior, decoupling has also been supported by neuroscientific research, showing that IDC is associated with a distinct pattern of brain activation including reduced activation of visual networks, and increased activation of the default mode network and lingual gyrus, reduced face processing, as well as increased EEG alpha activity (Benedek et al., 2011, 2014, 2016; Ceh et al., 2020, 2021; Denkova et al., 2018; Zhang et al., 2022a, b; for reviews in the context of creative cognition and imagination, see Benedek, 2018; Fink & Benedek, 2014). Recently, Cohen et al. (2022) showed that during an internal attentional focus the brain decouples from external stimuli as seen by opposing patterns of activity in six large-scale brain networks during external versus internal tasks. Furthermore, effects of PDec were also found in general motor performance, where it is studied in the context of the “constrained action hypothesis,” postulating that IDC results in a disruption of otherwise automatic motor processes (Dias da Silva & Postma, 2020; Dias da Silva & Postma, 2022; Kal et al., 2013; Kam et al., 2012; McNevin et al., 2003). For example, Dias da Silva and Postma (2022) showed that IDC negatively affects hand-tracking accuracy and increases velocity variation in a parallel visuomotor tracking task.

### Smooth pursuit eye movements

Smooth pursuit eye movements (SPEMs) refer to eye movements ensuring that a moving object is kept on the fovea (Lisberger, 2015). Due to the constant pursuit movement, they involve a uniform level of external attention over time, which enables a continuous assessment of eye behavior deviations over time. Therefore, SPEM is well suited for a time-critical analysis of PDec effects due to attentional engagement in a secondary task. Furthermore, ensuring accurate SPEM for extended time periods reflects a goal-directed activity involving sustained attention, and thus is considered a voluntary eye movement requiring more top-down control (Barnes, 2008) than the majority of eye parameters, for example, pupil diameter, blink rates, microsaccades, investigated in previous studies on IDC. We thus move from investigating global differences in eye behaviors between EDC and IDC to evaluating how a specific eye behavior (i.e., SPEM), imposed by a well-defined external task, is altered due to PDec elicited by internal task demands.

The accuracy of SPEM is commonly measured by velocity gain, defined as the ratio between gaze and target velocity, and the root mean square error (RMSE), reflecting the average absolute distance between gaze and target position over the pursuit period (Bargary et al., 2017; Hutton & Tegally, 2005; Kathmann et al., 1999; Lencer et al., 2019; Liversedge et al., 2013). Although RMSE has been widely used, Stubbs et al. (2018) argue that it does not adequately represent SPEM as it confounds the pursuit data with saccades, and it does not provide information about the accuracy of tracking speed. Further measures of SPEM performance reported in the literature include Euclidian gaze-to-target distance, the number of catch-up saccades (i.e., saccades decreasing the distance to the target), and anticipatory saccades (saccades aiming to land in front of the target; Bargary et al., 2017; Lencer et al., 2019; Liversedge et al., 2013; Stubbs et al., 2018). Several studies have already investigated SPEM in a dual task setting, although not with a focus of contrasting external and internal attention focus. Interestingly, Kathmann et al. (1999) found that simultaneous performance of an auditory discrimination task decreases pursuit error; however, later research refuted this finding, showing that a secondary task either does or does not affect SPEM performance (Hutton & Tegally, 2005; Kosch et al., 2018; Meyer et al., 2007; Sarac et al., 2021; Seya & Mori, 2015).

### The present study

Available research provides broad support for the involvement of PDec during IDC, which is especially evidenced by eye behavior becoming less determined by visual stimulation. Still, we do not understand well how PDec depends on the characteristics of the internal task. For example, does it involve a full-fledged decoupling response once a threshold is met or does it rather increase gradually with the level of internal demands? And is it more pronounced for visual compared to non-visual IDC, as the former might be prone to higher interference by external visual stimulation?

Therefore, the goal of the present study was to systematically evaluate effects of the internal task modality and of the level of internal demands on the degree of decoupling as assessed by SPEM accuracy. To this end, we examined how SPEM performance was affected by parallel performance of IDC tasks with either arithmetic or visuospatial material under two workload conditions. We generally assumed that external task performance (i.e., SPEM) becomes impaired by PDec elicited by engagement in the internal tasks. Considering task modality, we expected to see a higher degree of decoupling in the visuospatial compared to the arithmetic modality, as visuospatial working memory and spatial attention rely on common processing resources (Feng et al., 2012). Moreover, visuospatial IDC was shown to involve the coupling of eye behavior to internal processes (Johansson & Johansson, 2014), and cognitive performance was impaired when internal coupling was restricted (Damiano & Walther, 2019; Hale et al., 1996; Henderson et al., 2005; Lawrence et al., 2001; Pearson & Sahraie, 2003; Postle et al., 2006). Consequently, further interference can be expected when internal coupling during (visual) IDC is restricted due to the oculomotor EDC task. Moreover, we cautiously presumed that more versus less demanding internal tasks results in stronger PDec, based on one study showing that SPEM can be used to assess internal workload (Kosch et al., 2018), although other research has reported either mixed (Hutton & Tegally, 2005) or null findings associated with demand of the secondary task on SPEM (Meyer et al., 2007). Finally, we explore the temporal contingency of PDec effects to internal demands, and how sensitively they are captured by different SPEM measures.

## Method

### Open practices statement

This study was preregistered on the platform AsPredicted (https://aspredicted.org/B8P_MLZ), and any deviations from our preregistration are mentioned and explained in the article. We provide our materials, data, and analysis scripts on the Open Science Framework (OSF; https://osf.io/c256n/). Furthermore, we report power analysis, all manipulations and measures as well as data exclusion. Data preprocessing and analyses were performed with R (R Version 4.1.2, R Core Team, 2022) in RStudio (Version 2021.9.2.382, RStudio Team, 2020). Specific packages used are mentioned below.

### Power analysis

Sample size was determined a priori based on a power analysis using G*power version 3.1 (Faul et al., 2007). According to our previous research, we expected a within-subject effects to be around *dz* = 0.4 or higher (Annerer-Walcher et al., 2018, 2020, 2021). Alpha level was set to 0.05 to achieve a statistical power of 80%, which resulted in a required sample size of 41. To account for potential data exclusion, we recruited 50 participants.

### Participants

Data were collected between July and November in 2021. Fifty adults (36 female) aged 18–34 years (*M* = 22.86, *SD* = 2.78) participated in the experiment for payment (10 € per hour) or partial course credits. Most participants were students (96%). We do not expect deviations for the general population except perhaps in the elderly, as researched has shown that some ocular parameters decline with age (Dowiasch et al., 2015; Piquado et al., 2010). All participants had normal or corrected-to-normal vision (up to 0.5 diopter) and were native German speakers. Exclusion criteria included dyslexia, dyscalculia, problems distinguishing between left and right, neurological or psychological disorders, eye sickness, previous eye surgery, and active medications affecting eyesight or driving abilities. The study protocol was approved by the local ethics committee and all participants gave written informed consent.

This experiment was part of a larger test session, where participants also completed additional questionnaires and performed other independent tasks, that is not reported here. We provide additional data from the present experiment, including the Big 5 questionnaire, baseline pupil measures, and self-reported measures on the perceived internal load, on the study’s OSF (https://osf.io/c256n/). Data from the second task will become available after publication.

### Experimental design

This study employed a dual-task design, where participants performed an external, visual task (EDC; smooth pursuit) and in parallel performed an internal, mental task (IDC) under varying experimental conditions. To vary internal task modality, we implemented two different tasks: arithmetic and visuospatial. For the additional manipulation of internal workload, internal tasks were realized in two workload conditions (low and high) or not performed at all, thereby providing a control condition where participants only performed the external task (single-task control). All participants performed on all tasks and conditions, yielding a 2 (arithmetic, visuospatial) × 3 (control, low, high) within-subject design.

### Tasks and materials

#### External task

**Fig. 1:**
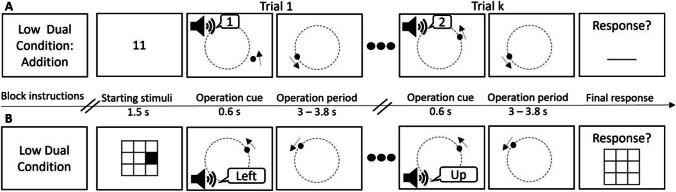
Sequence and timing of events in an experimental block. **A** Arithmetic internal task. **B** Visuospatial internal task. Stimuli are presented on a white background for readability; the original background color was grey (RGB 127, 127, 127). After the block instruction, a drift check and 2-s fixation cross were presented (not shown here). The internal task (A: arithmetic, B: visuospatial) then started with a starting stimulus (A: a number, B: a matrix with a colored patch), followed by 9–11 trials (10 on average) where single operation cues were presented denoting the operation to be carried out in the subsequent operation periods (A: mentally adding a number, B: mentally moving the patch in the indicated direction) in parallel to the smooth pursuit task (i.e., external task). At the end of each block, the final result had to be reported, before moving on with the next task block. The figure illustrates only the low internal workload condition of the respective task modalities. In the high-load condition, the arithmetic task required subtraction instead of addition of operations, and the visuospatial task required navigation through a 4 × 4 matrix instead of a 3 × 3 matrix. In the single condition, the smooth pursuit task was performed while ignoring the operation cues

The external task required continuous SPEMs. We presented participants with a black dot (RGB 0,0,0; radius 0.18 degrees of visual angle [°, dva]) on a grey background screen (RGB 127,127,127), which was moving on an imaginary circle (radius 5.4°) in a counterclockwise direction with 6.8 °/s starting at position 12 o’clock (see Fig. 1).

#### Internal tasks

Within each task modality, there were 30 blocks in which each of the workload conditions (control, low, high) was realized ten times. Each block consisted of 9–11 consecutive trials (ten on average), where a trial was defined by one internal operation. The sequence of blocks within each task modality was randomized but the order of trials in a block was fixed. A starting stimulus was presented at the beginning of a block, followed by on average ten trials where participants had to continuously perform the internal operations (always in respect to the result of a previous trial) until the end of the block, when they were prompted to report the final result of the internal operations. For example, in case of the arithmetic low workload condition, in one block participants had to add a number presented in Trial 1 to the starting number, remember the sum, add to it the number presented in Trial 2, etc., until the final trial, after which they completed the current block by reporting the final result (see below and Fig. 1). The specific settings of the workload conditions were determined based on extensive pilot tests, where we included an additional condition per task modality. We then chose the two conditions that differed in the performance rates and were at the same time neither too easy (participants might start mind wandering) nor too demanding (participants might get fatigued).

The *arithmetic task* was adapted from Buetti and Lleras (2016). It first showed a starting number on the screen that had to be memorized before it disappeared, and then a sequence of operation cues (i.e., numbers) was presented auditorily that either had to be added or subtracted mentally during the respective operation period (see Fig. 1A). In the *low workload condition*, participants started with a number between 10 and 40 and added 1 or 2 per trial; in the *high workload condition*, participants started with a number between 65 and 99 and subtracted 3 or 4 per trial. The starting numbers were chosen so that the intermediate and end results were smaller than 100 and larger than 10. In the blocks of the *single control condition*, the starting number was replaced by “XX,” and the same auditory cues were presented as in the dual-task conditions (half taken from low and half from high workload blocks, respectively), but they had to be ignored.

In the *visuospatial task*, participants had to mentally navigate through a square matrix similar to that in Kerr (1993); Fig. 1B). It first showed an initial matrix with one black patch indicating the starting location that had to be memorized before it disappeared, followed by a sequence of auditory operation cues (“left,” right,” up,” down”) indicating how the patch had to be moved mentally during the trial from its respective current position within the matrix. In the low workload condition, a 3 × 3 matrix (ca. 2.7° high and wide) was used, whereas in the high workload condition a 4 × 4 matrix (ca. 3.6° high and wide) was used to realize higher internal load. Operation cues were chosen so that the patch would not cross the outer border of the matrix, i.e., when the patch was next to the left border the next operation cue could not be “left.” Additionally, a constraint on the movements in opposite directions was imposed, for example, “right” could not be followed by “left,” “down” could not be followed by “up,” and vice versa. In the blocks of the single control condition, an empty matrix was presented (3 × 3 and 4 × 4 in half of the blocks, respectively) and the same operation cues as in the dual-task conditions were presented but had to be ignored.

Considering the complete design of the study, each task modality had three workload conditions, each consisting of ten blocks, which consisted of on average ten trials. This thus resulted in an overall 600 trials, i.e., 300 per task modality.

#### Procedure

All participants completed an online pre-screening survey to determine whether they met the study’s inclusion criteria. After participants arrived in our lab, their eyesight was confirmed with the Landolt vision test (Wesemann, 2002).

Each participant performed the dual-task paradigm for both internal tasks (arithmetic and visuospatial) with task order being counterbalanced across participants. Before the start of the task participants were given written instructions for both internal and external tasks (instructions were presented on-screen and printed out). After participants understood the tasks, they positioned their head on the chinrest and the eye tracker was calibrated.

The session started with four practice blocks (two control, one low and one high workload condition), where participants had to give the correct final response to the internal task in both the low and high workload conditions in order to continue to the main blocks. Overall, the test session lasted around 1.5 h, i.e., ca. 45 min per internal task, including a possible break after the completion of the first task.

Blocks followed a similar sequence for both internal tasks (Fig. 1). Each block started with a brief information about the workload condition. After participants confirmed having read the information by pressing the spacebar, a short drift check was applied, followed by a fixation cross (2 s), and the presentation of a starting stimulus for the respective internal task (1.5 s). Then, the external task (i.e., SPEM) started, and in parallel participants heard a sequence of auditory operation cues indicating the required internal operation of the respective internal task. Trial started with the operation cue, which lasted for 0.6 s and was followed by the operation period of 3–3.8 s, during which the participant had to perform the required internal operation. Intervals between trial onsets thus ranged randomly between 3.6 and 4.4 s (in 0.1-s increments, 4 s on average). This variability made operation cue onset less predictable and reduced a potential oculomotor inhibition preceding the expected auditory stimuli (Abeles et al., 2020). Furthermore, variable intervals also ensured that audio cues were randomly presented at different target positions, which decreases the influence of gaze position on pupil size (Steinhauer et al., 2022). At the end of a block, participants were prompted to report the final result of the internal task and then continue with the next block, or, in the single-task condition, skip to the next block. Participants were instructed that if they had lost track of operations, they should skip to the next block and not guess the result.

### Apparatus

The study took place in a sound-attenuated room protected from daylight and with lights on. The illuminance of the room, measured from the participant’s place, was 29.55 lx. Participants were seated in front of a 24-in. ASUS VG248qe monitor (1,920 × 1,080 pixels, ca. 33.52° × 19.73°, 60-Hz refresh rate, brightness and contrast set to 40% and 20%, respectively) and their heads were stabilized by a chin rest. The background of the display was grey (RGB 128,128,128), all letters and numbers were black (RGB 0,0,0; 0.37° high, font “Arial”). Sound was replayed through Logitech PC speakers Z 200 with the computer volume set to 100% and speaker volume at medium. Audio files were acquired from https://wideo.co/text-to-speech/ with voice “[de-DE] Lisa Fischer-S”. Mp3 files were converted to ogg files, for compatibility with PsychoPy, and edited to an equal length of 600 ms. The experimental paradigm was written in PsychoPy (Version 2020.2.10; Peirce et al., 2019).

Binocular eye-tracking data were acquired using the EyeLink 1000 Plus system (SR Research Ltd.) using Pupil-CR mode with a temporal resolution of 1,000 Hz. The setup was according to the manufacturer’s instructions: the chin rest was positioned centrally in front of the monitor at a distance of 88 cm so that participants’ line of sight was in the top quarter of the screen and the camera with illuminator was placed 59 cm in front of the chin rest. A 9-point calibration was conducted at the beginning of each task, controlled by the experimenter, with circles as targets on the default grey background. Calibration was validated and accepted as successful if the 9-point validation resulted in an average gaze error below 0.5° and maximum error below 1°, otherwise it was repeated until the desired thresholds were achieved. Drift checks were performed at the beginning of every block, and when the difference between gaze position and visual target was larger than 2° the participant was recalibrated.

### Data preprocessing

Eye-tracking data were exported using the EyeLink Data Viewer software package (SR Research Ltd., version 4.2.1). Dual-task blocks with missing answers (eight blocks from the arithmetic task, of which six were from the high workload condition) were excluded from the eye behavior analyses, as they included time periods when participants were not sufficiently focused on the internal task, and thus potentially eye behavior was related to the spontaneous rather than the goal-oriented IDC. Blinks were detected by the eye-tracking software and extended by 100 samples (100 ms) forward and backward to ensure that partial closure of the lid was also categorized as blink. Saccades were detected based on the average binocular velocity larger than 22 °/s and acceleration larger than 4,000 °/s^2^, according to EyeLink 1000 Plus User Manual (SR Research Ltd., version 1.0.18), with a minimum duration of 6 ms. Saccades with an amplitude smaller than 1° were labelled as microsaccades (McCamy et al., 2012). All data points not categorized as blinks or saccades were labelled as SPEM.

Gaze position (GP) and pupil diameter (PD) data were preprocessed separately. PD was transformed from arbitrary units to millimeters based on artificial pupil measures according to SR Research instructions. PD data were first smoothed with a moving average filter (n = 20) followed by a linear interpolation of missing data during blinks with the *gazeR* package (Geller et al., 2020). We excluded PD samples that were three standard deviations beyond an individual’s mean (0.38% of the whole dataset) and those recorded during saccades (4.33%) since PD cannot be reliably measured during a saccade. GP was transformed from pixels into dva to be used for SPEM and saccade analyses. Periods with blinks (7.73%), fixation disparity three standard deviations beyond an individual’s mean and larger than mean pupil distance (i.e., |60| mm; 0.98%) were excluded (Annerer-Walcher et al., 2021). We computed *gaze-to-target distance* (GTD) in terms of the Euclidean distance between gaze and target and excluded samples with a GTD larger than 6.8° (i.e., the distance the pursuit target travelled in 1 s, suggesting that participants completely lost track of the target). For *velocity gain* (VG) analysis, we excluded samples in fixations, i.e., gaze velocity smaller than 0.5 °/s, and calculated the ratio between gaze and target velocity, expressed in percentages. Values < 100 indicate that the eye moved slower than the target. Saccades were further categorized as anticipatory saccades (AS; increasing the distance to the target) or catch-up saccades (CUS; decreasing the distance to the target).

Each trial was binned into seven 0.5-s time-bins (i.e., 0–3.5 s relative to the trial start); samples beyond the seventh time-bin were discarded as our interstimulus interval (ISI) ranged between 3.6 and 4.4 s. A median PD (mm), VG (%), GTD (°) as well as the number of AS and CUS per second (Hz) were calculated per time-bin.

To ensure robust analyses, we excluded time-bins, trials, blocks, and participants with more than 50% of missing data due to any of the reported exclusion criteria (e.g., blinks, etc.). This resulted in the exclusion of 11.63%, 10.06%, and 2.08% of time-bins, 2.61%, 2.29%, and 0.61% of trials, and 1.30%, 1.10%, and 0.27% of blocks, from SPEM, saccade, and PD analyses, respectively. Finally, two participants had to be excluded from the GP-related analyses.

### Data analyses

Internal task performance, measured as a percentage of correct blocks, was analyzed with a 2 (task modality: arithmetic, visuospatial) × 2 (internal workload: low, high) within-subject analysis of variance (ANOVA).

Eye behavior data were analyzed with linear mixed models (LMMs) using the lmerTest package (Kuznetsova et al., 2017), where degrees of freedom are calculated using the Satterthwaite’s method. We fitted one model per eye parameter (PD, GTD, VG, CUS, and AS), which is a deviation from our preregistration, where we planned to fit three models per eye parameter (one per workload comparison). After data collection, we realized it is better to include workload as a fixed effect in one overall model instead of fitting three smaller models, as this allows for interaction tests between task modality, workload condition, and time-bin. The arithmetic task, the single task condition, and the first time-bin were considered as reference levels for the task modality, internal workload, and time-bin, respectively. Each model thus included the three-way interaction between the fixed factors (task, workload condition, time-bin), and order of the internal task (arithmetic task first or second, due to counterbalancing) as a fixed covariate, two random intercepts (participant and trial), and a random slope for internal task by workload condition. Here, fixed effects were used for experimental variables, which we manipulated; random intercepts per participant acknowledge that some participants respond differently than others (e.g., they pursue the target better resulting in smaller GTD) and random intercepts per trial reflect the fact that some trials are easier than others. Random slopes of internal task by workload condition represents the possibility that the effect of task and workload is not the same for all participants (e.g., some might find the arithmetic task more demanding than the visuospatial task, yet other might find subtraction less demanding than addition, etc.). We ran a principal component analysis on all the models (function “rePCA” from the lme4 package; Bates et al., 2015), which confirmed that the described random structure is supported by our data, i.e., each random component explained additional variance. Global fixed effects for a model were tested with a Type III ANOVA on the respective model, a function implemented into *lmerTest* package.

For the planned pairwise comparisons, we used the *emmeans* package (Lenth, 2022). This deviates from our preregistration, where we planned to use pairwise *t*-tests on the preprocessed data aggregated across trials. *Emmeans* uses the fitted model as an input, hence producing better estimations of the data. Instead of computing all possible pairwise comparisons, we compared the three workload conditions in each internal task and time-bin, since this was sufficient to answer our main research questions. Additionally, differences in PD between consecutive time-bins were evaluated for each workload and task condition. Effects related to internal task type were analyzed by computing the pairwise comparisons between internal tasks and workload conditions per time-bin. Bonferroni correction was applied to the group of tests within each time-bin (or workload condition and internal task combination in case of comparisons between time-bins), since an overall correction for more than 20 post-tests would be too conservative. Plots were produced by *emmeans* based on model data, showing estimated marginal means used for pairwise comparisons. No error bars are presented on plots, since the current approaches for confidence intervals in within-subject designs are not intended to be used with crossed random effects or are not yet adapted for random slopes and multifactorial design with crossed random effects (Politzer-Ahles, 2017). Readers are referred to examine confidence intervals of the pairwise comparisons instead of conditions’ means. For all comparisons we provide effect sizes using an approximation to Cohen’s *d* computed with the “eff_size” function from *emmeans,* which relies on the model’s residual degrees of freedom and estimated population *SD*. In LMMs with clustered design and more than one random factor, a sum of variances from random components can be used to estimate population *SD* (Hedges, 2007; Westfall et al., 2014). Readers are advised to interpret this effect size with caution, since the optimal calculation of effect sizes in LMMs is still debated (Rights & Sterba, 2019). Besides the estimate of the magnitude of effects, we also report Bayes factors (BFs) as an estimate of the weight of evidence in favor or against our hypotheses. BFs were computed with the *BayesFactor* package (Morey & Rouder, 2021) under a default Cauchy prior for each pairwise comparison based on the data aggregated across trials. BFs below 3 can be interpreted as weak, between 3 and 20 as moderate, between 20 and 150 as strong, and larger than 150 as very strong evidence (Wagenmakers, 2007).

## Results

### Manipulation check of workload conditions

**Fig. 2:**
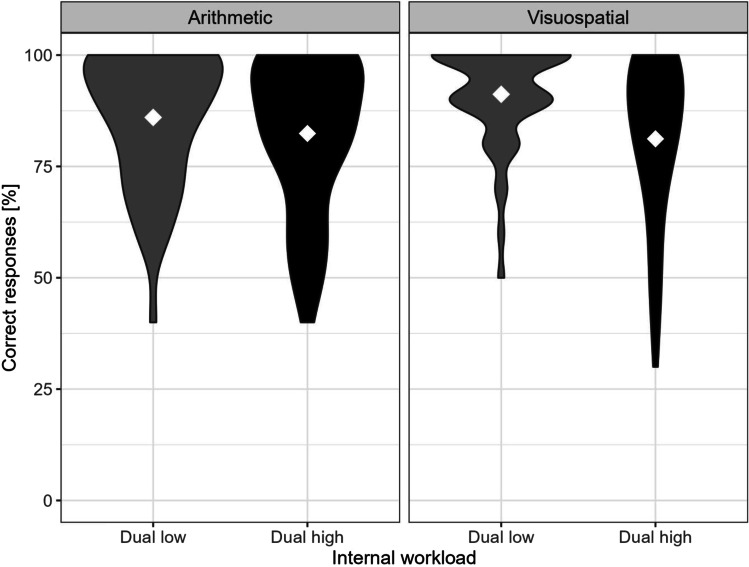
Distribution of correct responses for task modality (arithmetic, visuospatial) and dual workload conditions (low and high dual task). White diamonds represent condition means across trials and participants

The effectivity of the manipulation of workload conditions was examined in terms of task performance (correct responses) between low and high conditions in both internal tasks (arithmetic and visuospatial). Figure 2 shows the distribution of correct responses for both task modalities and dual workload conditions. A within-subject ANOVA comparing task performance yielded a significant main effect of workload (*F*(1, 48) = 17.78, *p* < .001, *η*2 = .05), indicating that solution rates were higher in the low compared to the high conditions (arithmetic: 86%, *SD* = 14.71% vs. 82.4%, *SD* = 17.1%; visuospatial: 91.2%, *SD* = 11.18%, versus 81.2%, *SD* = 18.37%), while the main effect of task type and the interaction were not significant, *F*(1, 48) = 1.99, *p* = .164, *η*2 = .01 and *F*(1, 48) = 3.52, *p* = .067, *η*2 = .01, respectively.

**Fig. 3:**
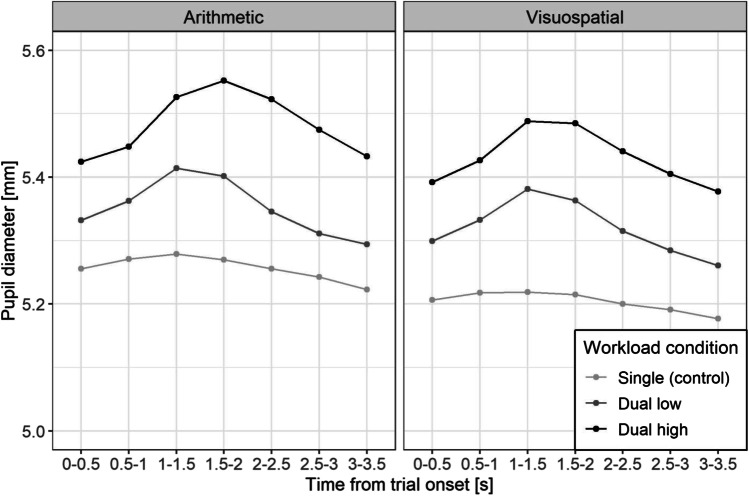
Pupil diameter during smooth pursuit eye movements (SPEMs) for task modality (arithmetic, visuospatial) across the trial duration for all workload conditions (single task, low and high dual task). For readability statistical significance is not depicted since both dual tasks are significantly different from the control throughout the trial

Additionally, we analyzed PD as a physiological index of internal workload for all three workload conditions (single task, low, and high). Internal workload is known to evoke a small but robust pupil dilation due to task relevant changes in attentional effort and arousal (Beatty & Lucero-Wagoner, 2000). Stimulus-evoked PD response thus corresponds to the phasic arousal, which indicates internal workload, while baseline PD corresponds to the tonic arousal, which indicates the general arousal state (Cohen Hoffing et al., 2020). Effects of workload conditions on PD were examined with a LMM (see Table 1 for global effects and Table A.1 in the Online Supplemental Material (OSM) for model summaries). Planned pairwise comparisons of workload conditions showed significant PD differences between all workload conditions (high > low > control) at all time points in both tasks, supported by BFs showing strong to very strong evidence for these claims (Fig. 3, Table A.2). The consistent difference between workload conditions across all time-bins indicates tonic PD response and implies that attentional effort was increased throughout a block of low and high trials, i.e., it returned to the block-level baseline but never reached the control condition level. Effects of workload were very similar in both internal tasks (arithmetic and visuospatial; Table A.3). Time-based comparison further showed phasic PD response in the two dual-task conditions, reaching its peak in the third time-bin (or fourth in the case of a high arithmetic task), and decreased after the fourth time-bin (Fig. 3, Table A.4). Differences between time-bin in control conditions were not supported by frequentist post-tests (suggesting no internal load); however, BFs captured evidence against the null hypothesis (Table A.4).

**Table 1:** Type III ANOVA table with Satterthwaite’s method for the linear mixed models showing global effects of the selected eye parameters

Eye parameter	Effect	*Sum sq.*	*Mean sq.*	*DF*_*num*_	*DF*_*den*_	*F*	*p*
Pupil diameter [mm]	Task	0.15	0.15	1	51.06	1.02	.318
	Load	9.51	4.75	2	80.60	32.52	< .01
	Time	197.15	32.86	6	204498.20	224.78	< .01
	(Task) order	0.06	0.06	1	48	0.42	.521
	Task×Load	0.11	0.05	2	119.12	0.37	.691
	Task×Time	2.66	0.44	6	204498.20	3.03	< .01
	Load×Time	54.68	4.56	12	204498.10	31.17	< .01
	Task×Load×Time	5.14	0.43	12	204498.10	2.93	< .01
Gaze-to-target distance [dva]	Task	1.67	1.67	1	48.92	4.7	.035
	Load	4.51	2.25	2	58.97	6.37	< .01
	Time	64.56	10.76	6	179613.20	30.38	< .01
	(Task) order	0.12	0.12	1	46.03	0.35	.558
	Task×Load	5.45	2.72	2	69.47	7.69	< .01
	Task×Time	9.93	1.65	6	179614.70	4.67	< .01
	Load×Time	54.67	4.56	12	179614.70	12.86	< .01
	Task×Load×Time	10.25	0.85	12	179617.60	2.41	< .01
Velocity gain [%]	Task	1027.42	1027.42	1	50.98	3.36	.073
	Load	4811.96	2405.98	2	62.49	7.87	< .01
	Time	128333.40	21388.89	6	179632.40	69.93	< .01
	(Task) order	721.38	721.38	1	45.99	2.36	.131
	Task×Load	5007.51	2503.76	2	88.82	8.19	< .01
	Task×Time	15669.92	2611.65	6	179636.10	8.54	< .01
	Load×Time	112860.70	9405.06	12	179636.10	30.75	< .01
	Task×Load×Time	11734.20	977.85	12	179638.60	3.20	< .01

*Note.* Interaction between factors is represented with the multiplication sign. DF_num_ = degrees of freedom of the numerator; DF_den_ = degrees of freedom of the denominator; dva = degrees of visual angle; load = workload condition (single, dual low and high); time = time-bin; task = internal task (arithmetic, visuospatial); (Task) order = consecutive order of the internal task

### Effects of internally directed cognition on smooth pursuit eye movement

#### Direct smooth pursuit eye movement measures

**Fig. 4:**
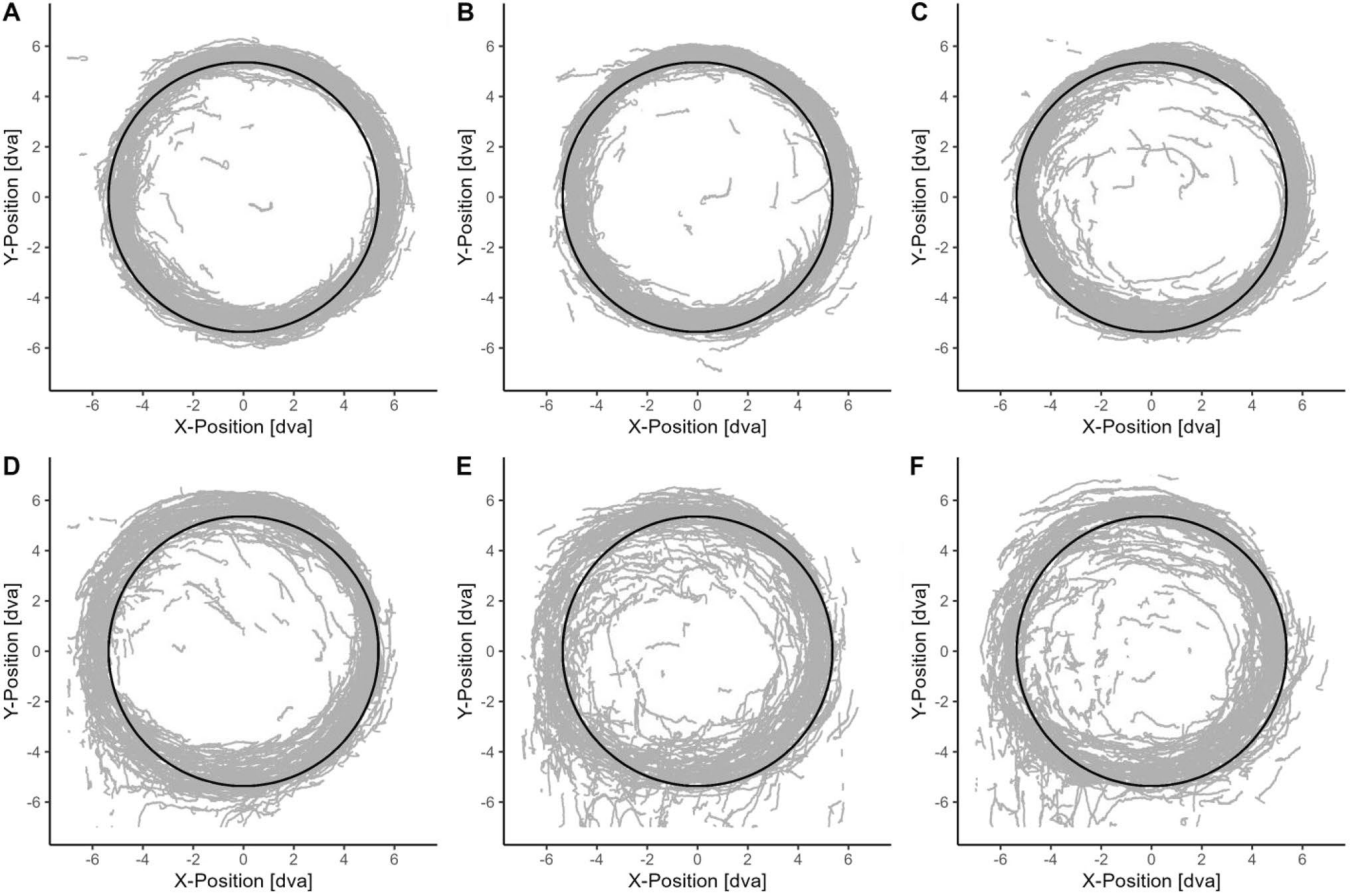
Smooth pursuit trajectory and eye gaze traces of a random subject for different task modalities (arithmetic, visuospatial) and all workload conditions (single task, low and high dual task). A random subject’s gaze traces (grey) following target trajectory (black) across all blocks in a respective condition. **A–C** arithmetic task, **D–F** visuospatial task. Left to right: single task, low dual, and high dual task. Dva = degrees of visual angle

**Fig. 5:**
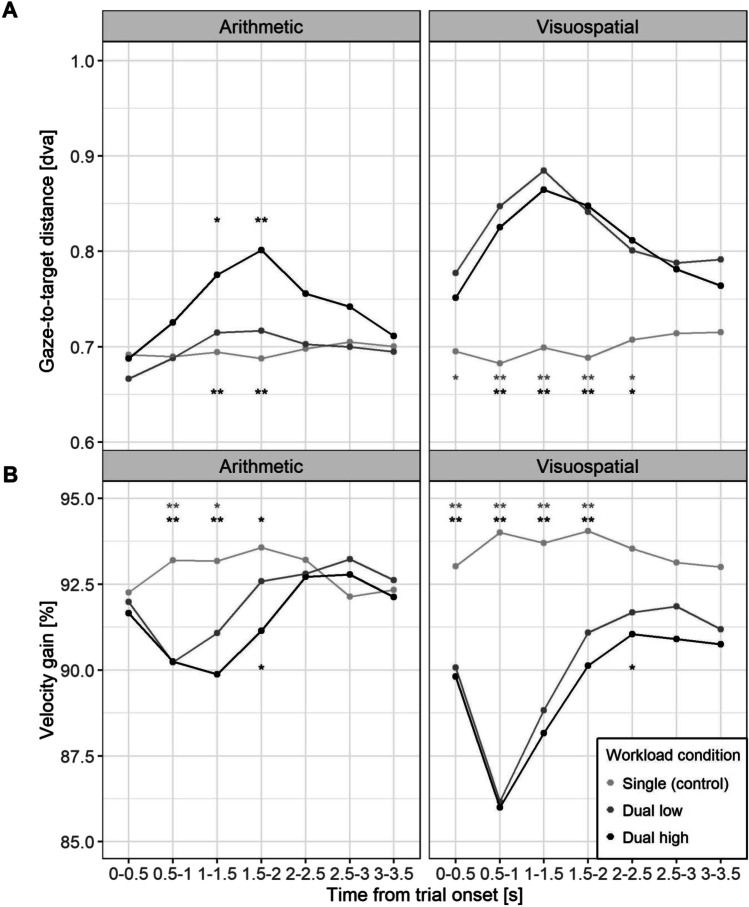
Gaze-to-target distance (**A**) and velocity gain (**B**) during smooth pursuit eye movements (SPEMs) for different task modalities (arithmetic, visuospatial) across the trial duration for all workload conditions (single task, low and high dual task). Statistically significant differences are indicated with asterisks (* *p* < .05; ** *p* < .01). Asterisks near the single condition line represent significant differences between single vs. low condition (top row, grey) and single vs. high condition (bottom row, black). Asterisks near the high condition line represent significant differences between low vs. high condition (black). Dva = degrees of visual angle

Figure 4 shows the target trajectory and raw eye movement traces of a single block performed by a random participant. Accuracy of pursuit during SPEM was assessed with two direct measures, gaze-target-distance (GTD) and velocity gain (VG). A perfect pursuit tracking would result in a 0° GTD, i.e., gaze and target position is exactly the same, and a VG of 100%, i.e., eyes are moving with the exact same speed as the target; thus, higher GTD and deviations in VG indicate PDec. Indeed, GTD and VG were clearly affected by additional performance of internal tasks compared to single task control conditions across both task modalities as evidenced by significant two-way and three-way interactions involving workload (Fig. 5, Table 1). Frequentist and Bayesian inference were in agreement across all comparisons (Table 2, Tables A.2 and A.3). Summaries of the main models are given in Table A.1. Significant pairwise comparisons are shown in Table 2, and a complete report including standard errors and confidence intervals of the estimate can be found in Table A.2. Additionally, VG was calculated and analyzed using angular velocity (see OSM B, Fig. B1).

**Table 2:** Planned pairwise comparisons of task modality (arithmetic vs. visuospatial) by internal workload contrasts

Eye parameter	Workload comparison	Time	*Estimate*	*t*	*p*	*Effect size*	*BF10*	*BF01*
Gaze-to-target distance [dva]	Single – Low	0–0.5	0.11	3.07	< .01	0.14	39.89	0.03
	Single – High	0.5–1	0.11	2.87	.015	0.14	14.96	0.07
	Low – High	1–1.5	-0.08	-2.64	.027	-0.11	3.11	0.32
	Single – Low	1.5–2	0.12	3.52	< .01	0.16	52.47	0.02
	Low – High	1.5–2	-0.08	-2.55	.035	-0.10	2.09	0.48
	Single – Low	2–2.5	0.09	2.54	.037	0.12	4.87	0.21
Velocity gain [%]	Single – Low	0–0.5	-2.67	-3.31	< .01	-0.12	454.76	< .01
	Single – High	0–0.5	-2.60	-2.76	.020	-0.12	29.85	0.03
	Single – Low	1–1.5	-2.77	-3.41	< .01	-0.13	47.32	0.02
	Single – Low	1.5–2	-1.98	-2.44	.047	-0.09	21.10	0.05
	Single – Low	2.5–3	-2.36	-2.92	.012	-0.11	30.06	0.03
	Single – High	2.5–3	-2.86	-3.03	< .01	-0.13	9.33	0.11
	Single – Low	3–3.5	-2.09	-2.56	.033	-0.10	11.90	0.08

*Note.* Only statistically significant comparisons are shown. *BF* = Bayes factor; *BF*_*10*_ = ratio of evidence in favor of effect; *BF*_*01*_ = ratio of evidence against effect; dva = degrees of visual angle; low = low dual task; high = high dual task; single = single task

In the arithmetic task, both GTD and VG differ from control from around 0.5 to 2 s after operation cue (see Fig. 5), corresponding to the expected duration of the internal operation as evidenced by the dynamics of PD (see Fig. 3). Internal workload thus elicited changes in both direct SPEM measures, consistent with the assumption of PDec. For GTD we observed clear differences between the low and high conditions, whereas the low condition did not differ from the control (Fig. 5A, Table A.2). In contrast, VG was affected similarly in both workload conditions at early time points and only at a later point the high condition resulted in slower pursuit. This indicates a prolonged PDec effect for the more demanding (high) internal task.

In visuospatial task, low and high conditions resulted in substantial increases of GTD and decreases of VG almost throughout the whole trial duration, except for the last two time-bins (Fig. 5, Table A.2). Interestingly, effects on GTD and VG were very similar for low and high conditions in the visuospatial task; the only difference between workload conditions was again observed with VG at a later time in the trial.

Workload effects on VG and GTD were moderated by task modality, reflecting more pronounced effects in the visuospatial compared to the arithmetic task (see Table 2 for statistically significant comparisons, and Table A.3 for the complete report). Specifically, in the visuospatial task, effects started earlier, had a higher peak, and were more sustained compared to arithmetic task. Yet, workload differences between the low and high condition appeared more consistent in the arithmetic task. Overall, the split of attentional focus between internal and external tasks clearly impaired the execution of SPEM, suggesting effects of PDec, with effects being moderated by task modality and level of internal workload.

#### Indirect smooth pursuit eye movement measures

**Fig. 6:**
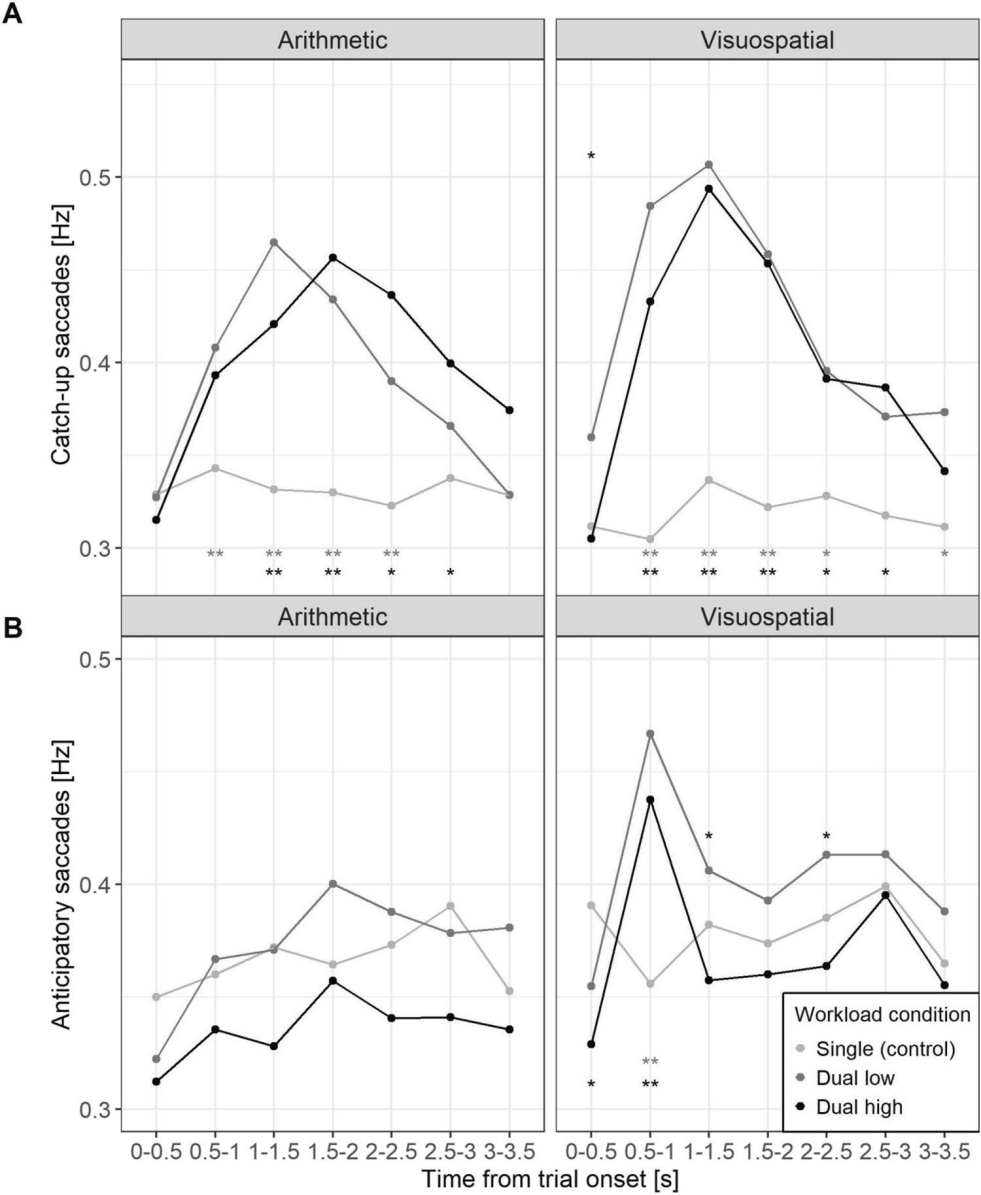
Catch-up-saccades (**A**) and anticipatory saccades (**B**) during smooth pursuit eye movement measures (SPEMs) for task modality (arithmetic vs. visuospatial) across the trial duration for all workload conditions (single task, low and high dual task). Statistically significant differences are presented with asterisks (* p < .05; ** p < .01). Asterisks in the bottom section represent significant difference between the single vs. low condition (first row, grey) and single vs. high condition (bottom row, black). Asterisks in the topsection present significant difference between low vs. high condition

Main SPEM assessment was complemented by two indirect measures, catch-up saccades (CUS), and anticipatory saccades (AS). We had no prediction about the direction of the effects of task modality and workload on these two measures. Results show that CUS were affected similarly in both modality and workload conditions, while the number of AS was only altered in the visuospatial task with some differences between low and high workload condition (Fig. 6, Table A.1). The global effects followed the same pattern with a statistically significant three-way interaction involving workload for AS and CUS (Table A.5). Pairwise comparisons are given in Table A.2 and A3. *T-*tests and BFs were mostly in agreement across all comparisons. Additionally, CUS and AS were classified and analyzed using angular degrees, see OSM B (Fig. B2).

CUS increased during dual-task conditions relative to the single (control) task in both task modalities for the majority of the trial duration (Fig. 6A, Table A.2). There were generally no differences between low and high conditions in either arithmetic or visuospatial task (except for CUS in the first time-bin of the visuospatial task). Slight differences between task modalities can be observed around 1 s after operation cue onset and at the end of the trial, where more CUS were made in visuospatial compared to arithmetic task (Table A.3). Overall, the division of cognitive resources between the external and internal task led to decoupling of attentional resources from the external task, which resulted in less accurate SPEMs (Fig. 5) and compensatory eye behavior as evidenced by more CUS (Fig. 6A).

For AS, IDC effects were only observed in the visuospatial task (Fig. 6B). Interestingly, there was a relatively large increase of AS compared to control right after the operation cue in the visuospatial task. One possible explanation for this effect could be related to internal coupling, suggesting that participants made a saccade in the direction indicated by audio instruction (i.e., up, left, etc.). A brief exploratory post hoc investigation revealed some support for this notion, although the effect was not fully consistent across the four cardinal directions, see OSM B (Fig. B3).

Interestingly, in the visuospatial task we observed higher CUS and AS in the low compared to high condition. While this was not predicted, it could suggest that there were more cognitive resources left for the planning of saccades, which especially in case of AS require predicting the target’s trajectory. Taken together, indirect SPEM revealed further support of PDec effects, especially in terms of increased numbers of catch-up saccades in response to both task modalities.

## Discussion

In the present study, we systematically investigated how perceptual decoupling (PDec) depends on internally directed cognition (IDC). The study specifically investigated the effects of internal task modality (arithmetic vs. visuospatial) and workload (low vs. high) on the accuracy of smooth pursuit eye movements (SPEMs) relative to a control condition. We found that IDC substantially impaired SPEM performance as evidenced by higher gaze-to-target distance (GTD), lower velocity gain (VG), and a higher number of catch-up saccades (CUS) as well as changes in anticipatory saccades (AS), and results were moderated by task modality and workload condition. We now discuss how these findings inform our understanding of the PDec phenomenon.

### Effects of task modality on perceptual decoupling

As predicted, the internal visuospatial task interfered with SPEM more than the arithmetic task, as seen in most SPEM parameters. However, these differences were neither observed in PD nor in behavioral measures, where effects were comparable across both task modalities, suggesting that the internal spatial navigation through a matrix is not per se more demanding than performing mental arithmetic. This result pattern suggests that visuospatial IDC yields higher interference with external, visual attention than arithmetic IDC, as they share more similar resources in (visual) working memory. Indeed, spatial attention plays an important role in visuospatial working memory (Feng et al., 2012), especially during the maintenance and manipulation of location information (Awh & Jonides, 2001). Importantly, interference between external and internal visuospatial tasks may not only arise from processing similar content, but also at the level of associated eye movements, as eye behavior tends to couple to characteristics of internal visuospatial tasks (Johansson & Johansson, 2014). Studies found that restrictions to eye movements impair visuospatial, but not verbal working memory retention (Hale et al., 1996; Lawrence et al., 2001; Pearson & Sahraie, 2003; Postle et al., 2006). Another study comparing eye behavior between various EDC and IDC tasks (i.e., verbal, arithmetic, and visuospatial) found that eye behaviors associated with external and internal arithmetic and verbal tasks were highly consistent, but very different for visuospatial tasks, again suggesting that external and internal visuospatial tasks both have independent effects on eye behavior (Annerer-Walcher et al., 2021). Taken together, this interference effect indicates that PDec is sensitive to the modality of IDC. PDec increases when internal and external tasks rely on similar (visuospatial) attentional resources implying a higher need to decouple ongoing internal processes from visual sensory information processing.

### Effect of workload on perceptual decoupling

Manipulation checks confirmed the presumed workload differences between low and high workload conditions for both task modalities (i.e., arithmetic and visuospatial). As expected, higher workload resulted in lower task performance. Moreover, the high workload condition yielded higher PD than the low workload condition, which again had higher PD than the single task control condition, and PD differences were clearly contingent to the onset of the operation cue and thus the time-course of the internal task performance. As workload demands increase the activity in the locus coeruleus norepinephrine system, which in turn increases PD (van der Wel & van Steenbergen, 2018), the reported pupil responses support the effective manipulation of workload differences in IDC.

Internal workload effects on SPEM performance showed some evidence for a gradual PDec response, i.e., increasing internal demands resulted in lower SPEM performance and thus increased PDec. This finding suggests that PDec is related to the amount of internal resources used. The gradual PDec response was especially evident in the arithmetic task, where SPEM were impaired more and for longer time under high compared to low workload, indicating a higher degree of PDec. The visuospatial task evoked overall strong effects on SPEM (stronger than the hard arithmetic task), but surprisingly no significant differences between the workload conditions. One possible explanation could be related to the fact that, in the visuospatial task, workload conditions differed with regard to the grid size that had to be considered in the internal operation, but besides that required the same internal operation, i.e., moving one step in one cardinal direction. This is quite different from the arithmetic task, where the operations themselves differed between low and high workload (addition vs. subtraction), while the intermediate result to be memorized was largely similar. It thus is possible that SPEM performance (GTD and VG) and thus PDec is more sensitive to the operation processing, but less to the memory component of the task (that may have been reflected in pupil diameter). This points towards an interesting differentiation of how working memory was taxed (i.e., manipulation vs. retention; cf. Sauseng et al., 2005), and to associated differences in the specificity of eye parameters.

Another potential explanation for the overall strong impairment of SPEM but lack of workload differences in the visuospatial task is a ceiling effect in the PDec response. Potentially, PDec increases gradually up to a certain degree, but once a certain level of visual working memory load is reached, the internal task is prioritized and PDec is fully engaged. Since the visuospatial task and SPEM require similar resources (see previous section), a lower IDC workload might already impose the maximum demand on shared visuospatial resources. Any higher workload then might not cause PDec to further increase as it is already fully engaged. Moreover, maintaining visuospatial information in working memory would draw additional attentional resources independent of operation processing. The observed direct SPEM effects (GTD and VG) offer some support for this notion, as both low and high workload conditions differed from the control condition already at the operation cue onset and never reached the level of the control condition throughout the trial, suggesting that simply keeping the matrix in working memory results in interference with SPEM.

Lastly, it is also possible that the workload in the easier arithmetic task was too low to elicit any eye behavior changes. In fact, the low condition did not differ from the control with respect to GTD, but only for VG, showing that VG was generally more sensitive than GTD in detecting workload differences. Here, it needs to be considered that the positional lag behind the target (i.e., GTD) can only be detected after eyes have decelerated (i.e., VG) for some time, and thus GTD effects may not be observed when the deceleration in the low condition did not last long enough.

Interestingly, findings for workload variations have been rather inconsistent in the SPEM literature so far. Kosch et al. (2018) observed higher impairments of SPEM for increased load in mathematical n-back tasks. On the other hand, Hutton and Tegally (2005) reported mixed findings for different dual-task conditions: spatial tapping compared to single key tapping resulted in significant impairment of SPEM, but no differences emerged between random number generation and sequential counting from one to ten. Finally, investigating SPEM during backward counting in steps of seven and 13, Meyer et al. (2007) reported decreased VG for both workload levels but no difference between them. Importantly, while most studies used a horizontally moving SPEM target, Kosch et al. (2018) compared three target trajectories, i.e., rectangle, circle and sine, and reported different effects of a secondary task on SPEM depending on the target trajectory. They showed that circular and sinusoidal trajectories require more effort than rectangular, as seen by higher gaze deviations, which could explain some of the mixed findings from the literature.

Taken together, we only see some support for a graded PDec response, that is, that PDec reflects the amount of workload. It seems possible that PDec may run into ceiling effects, or that certain SPEM parameters were either not sensitive enough to capture subtle workload differences, or are actually specific to the ongoing internal operation (i.e., memory manipulation vs. retention; cf. Sauseng et al., 2005).

### Time-course of perceptual decoupling

Our design enabled us not only to describe the effects of internal task modality and workload on a general level, but it also allowed us to tap into the dynamic evolvement of PDec over time. The measures of GTD, VG, and CUS showed that the degree of PDec is clearly dependent on the moment-by-moment demands of the internal operation imposed by the respective task. Operation time appeared to last about 1–2 s (and tended to be longer for higher load). Specifically, all measures resulted in a similar pattern across workload and task modality. In the dual-task conditions, PDec started increasing after operation cue onset and returned towards the level of control in the second half of the trial. PDec is thus time-contingent to the internal task, reflecting a division of cognitive resources between IDC and EDC limited to the time of increased internal demands.

Importantly, although participants were aware that their eyes were being tracked, the time-course of PDec might have been affected by the prioritization of the internal task, as the internal task required an active response at the end of the block whereas the external task did not. In the current study participants were not given any instructions regarding task priority, thus, instruction to prioritize the external task could have led to reduced SPEM impairments. For example, attention might not be able to decouple from the perceptual (visual) input, when the main priority is placed on timely visual processing and accurate oculomotor response. Since both tasks share common cognitive resources, prioritizing the external task would then presumably result in decreased performance of the internal task (Verschooren et al., 2019). Nonetheless, it is possible that even though a higher priority would be placed on the SPEM, internal task performance would still elicit some degree of PDec. According to Smallwood and Schooler (2015), it is yet unknown whether PDec plays a functional role in suppressing external information processing to shield the internal thought or it is merely a consequence of limited cognitive resources reflecting the lack of attentional focus towards the external stimuli. Future research manipulating the task priority could hence advance our understanding of the functional role of PDec.

Although SPEM per definition cannot include saccades, research has started to acknowledge the synergy between the both ocular systems and some even argue for a single sensorimotor process (Goettker & Gegenfurtner, 2021; Orban de Xivry & Lefevre, 2007). Traditionally SPEM and saccades were thought to be exclusively mediated through a motion and position pathway, respectively, however, more recent research has shown that a collaborative response of SPEM and saccades during target tracking is based on shared motion and position information (Goettker, Braun, & Gegenfurtner, 2019a; Goettker, Brenner, et al., 2019b; Orban de Xivry et al., 2006). Our results of SPEM and CUS showed that dual-task conditions with less accurate pursuit also included a higher number of saccades, suggesting a close relationship between the two ocular systems. While the increased number of CUS could merely be a consequence of increased position lag due to PDec, it could also suggest a shared information stream and synergistic action between SPEM and saccades.

Inspection of AS did not show the same time-course as the other SPEM parameters. However, in the visuospatial task, we observed a strong increase in AS right after the operation cue onset in both workload conditions. Interestingly, at the same time point a large deceleration was observed in VG, which could point towards a general strategy for dual task performance. Participants made an anticipatory saccade to overtake the target and then slowed down, thus freeing resources for the performance of the visuospatial task. The anticipatory saccade would allow them to reduce the pursuit speed, while they were internally navigating through the matrix, without losing sight of the target. Another possible explanation would be the effect of internal coupling, that is, participants would make a saccade in the direction of the spatial cue (Spivey & Geng, 2001). A brief post hoc exploratory analysis showed some support for this effect (Figure B). Examination of anticipatory saccades from the second time-bin of the dual-task condition (0.5–1 s), pooled across low and high workload, in relation to the operation direction (left, right, up, down) showed that in the “right” trials the direction of saccades was mostly right, i.e., within the 45° around 0°. This effect was also to a lesser extent present in the “up” and “down” trials, where most of the saccades were made in a wider (~180°) up and down direction, respectively. Lastly, saccades in the “left” trials were made in both horizontal directions, but there was no clear bias to the left side apparent. Together the results are not fully conclusive in supporting an internal coupling effect, which may be partly due to the fact that guided (circular) eye movements imposed by the SPEM task may have obscured the internal coupling of saccades. Future studies should examine internal coupling effects in more appropriate settings.

### Limitations and future directions

One particularly interesting finding of this study was the observation of a differential effect of workload on different eye parameters. While workload conditions resulted in consistent task performance differences for both internal tasks, it is possible that these differences partly resulted from how internal tasks recruited working memory in terms of manipulation versus retention aspects. We speculated that PDec, in contrast to PD, might be specifically sensitive to working memory manipulation and less to working memory load in general, which awaits replication in devoted future studies. As another curious finding, our explorations of AS suggested the occurrence of internal coupling during the visuospatial task, which may actually have strengthened PDec effects due to discrepancy of eye behaviors imposed by external and internal tasks (SPEM vs. following the direction of the operation cue). Yet, future studies should try to vary internal coupling demands independent of task modality, to disentangle interference effects of task modality and internal coupling. While this study focused on SPEM allowing continuous assessments of deviations from expected eye behavior, PDec effects should also be investigated systematically for other voluntary eye movements to establish the robustness and generalizability of our findings. Moreover, further work could also consider exploring whether PDec effects generalize even to reflexive eye behaviors or are limited to voluntary eye movements. Together these analyses will help us to understand whether eye behavior changes are merely a consequence of a largely automatic decoupling of attention due to limited resources or may play a more active role in the shielding of IDC from visual distraction.

## Conclusion

We have shown that IDC impairs visual processing during SPEM with effects being moderated by the modality, workload, and time-course of IDC. We interpret these findings as evidence in support of the PDec mechanism that facilitates goal-directed IDC by decoupling attention from interfering visual input. The findings elucidate specific functions of the PDec mechanism by showing that it is specific to the time-course and degree of internal load and particularly strong when internal processing relies on the same (visual) modality as the external task. The consideration of multiple indicators of SPEM performance offered detailed insight in how external performance was impaired by different forms of IDC. The findings further suggested that different eye parameters are sensitive to different aspects of cognitive workload, offering time-critical information beyond PD, and thus together serve as objective indicators of internal attention focus.

## Supplementary Information

ESM 1 (PDF 344 kb)
