# Supplementary material for: Effects of internally directed cognition on smooth pursuit eye movements: A systematic examination of perceptual decoupling
Source: Atten Percept Psychophys. 2023 Mar 15;85(4):1159–78. doi: 10.3758/s13414-023-02688-3 (PMC10167146; doi:10.3758/s13414-023-02688-3)
Supplement: Supplementary file 1 — (PDF 344 kb) [file 13414_2023_2688_MOESM1_ESM.pdf]

	Pupil diameter [mm]			Gaze-to-target distance [dva]		
Predictors	Estimates	*SE*	*t*	Estimates	*SE*	*t*
(Intercept)	5.27	0.11	46.55	0.70	0.04	19.06
task [vs]	-0.05	0.05	-1.02	0.00	0.04	0.10
load [easy]	0.08	0.03	2.97	-0.03	0.02	-1.22
load [hard]	0.17	0.03	5.32	0.00	0.02	-0.15
time0.5-1	0.02	0.01	1.97	0.00	0.01	-0.14
time1-1.5	0.02	0.01	3.03	0.00	0.01	0.22
time1.5-2	0.01	0.01	1.85	0.00	0.01	-0.31
time2-2.5	0.00	0.01	-0.01	0.01	0.01	0.51
time2.5-3	-0.01	0.01	-1.72	0.01	0.01	1.06
time3-3.5	-0.03	0.01	-4.22	0.01	0.01	0.69
taskOrder [2]	-0.03	0.04	-0.65	-0.01	0.03	-0.59
task [vs] * load [easy]	0.02	0.03	0.53	0.11	0.04	3.07
task [vs] * load [hard]	0.02	0.04	0.45	0.06	0.04	1.62
taskvs:time0.5-1	0.00	0.01	-0.37	-0.01	0.02	-0.59
taskvs:time1-1.5	-0.01	0.01	-1.04	0.00	0.02	0.06
taskvs:time1.5-2	-0.01	0.01	-0.55	0.00	0.02	-0.16
taskvs:time2-2.5	-0.01	0.01	-0.60	0.01	0.02	0.30
taskvs:time2.5-3	0.00	0.01	-0.18	0.01	0.02	0.30
taskvs:time3-3.5	0.00	0.01	0.31	0.01	0.02	0.62
loadeasy:time0.5-1	0.00	0.01	1.42	0.02	0.02	1.31
loadhard:time0.5-1	0.01	0.01	0.78	0.04	0.02	2.18
loadeasy:time1-1.5	0.06	0.01	5.35	0.05	0.02	2.53
loadhard:time1-1.5	0.08	0.01	7.17	0.08	0.02	4.71
loadeasy:time1.5-2	0.06	0.01	5.08	0.05	0.02	3.03
loadhard:time1.5-2	0.11	0.01	10.40	0.12	0.02	6.49
loadeasy:time2-2.5	0.01	0.01	1.23	0.03	0.02	1.65
loadhard:time2-2.5	0.10	0.01	8.98	0.06	0.02	3.39
loadeasy:time2.5-3	-0.01	0.01	-0.72	0.02	0.02	1.11
loadhard:time2.5-3	0.06	0.01	5.80	0.04	0.02	2.26
loadeasy:time3-3.5	-0.01	0.01	-0.45	0.02	0.02	1.07
loadhard:time3-3.5	0.04	0.01	3.72	0.01	0.02	0.80
taskvs:loadeasy:time0.5-1	0.01	0.02	0.43	0.06	0.03	2.29
taskvs:loadhard:time0.5-1	0.01	0.02	0.95	0.05	0.03	1.84
taskvs:loadeasy:time1-1.5	0.01	0.02	0.75	0.06	0.03	2.27
taskvs:loadhard:time1-1.5	0.01	0.02	0.36	0.02	0.03	0.96
taskvs:loadeasy:time1.5-2	0.00	0.02	0.00	0.02	0.03	0.64
taskvs:loadhard:time1.5-2	-0.03	0.02	-1.91	-0.01	0.03	-0.56
taskvs:loadeasy:time2-2.5	0.01	0.02	0.59	-0.02	0.03	-0.71
taskvs:loadhard:time2-2.5	-0.04	0.02	-2.78	-0.01	0.03	-0.52
taskvs:loadeasy:time2.5-3	0.01	0.02	0.51	-0.03	0.03	-1.12
taskvs:loadhard:time2.5-3	-0.04	0.02	-2.29	-0.03	0.03	-1.17
taskvs:loadeasy:time3-3.5	0.00	0.02	-0.28	-0.03	0.03	-0.99
taskvs:loadhard:time3-3.5	-0.03	0.02	-1.71	-0.02	0.03	-0.86
**Random Effects**						
σ^2	0.15			0.35		

For Review Only

τ_{00}	0.01 trial<br>0.61 participant	0.00 trial<br>0.05 participant
τ_{11}	0.10 participant.taskvs<br>0.02 participant.loadeasy<br>0.04 participant.loadhard<br>0.02 participant.taskvs:loadeasy<br>0.05 participant.taskvs:loadhard	0.05 participant.taskvs<br>0.01 participant.loadeasy<br>0.02 participant.loadhard<br>0.04 participant.taskvs:loadeasy<br>0.04 participant.taskvs:loadhard
ρ_{01}	-0.20 participant.taskvs<br>-0.02 participant.loadeasy<br>0.09 participant.loadhard<br>0.10 participant.taskvs:loadeasy<br>0.18 participant.taskvs:loadhard	-0.39 participant.taskvs<br>-0.03 participant.loadeasy<br>0.12 participant.loadhard<br>0.62 participant.taskvs:loadeasy<br>0.42 participant.taskvs:loadhard
ICC	0.82	0.19
N	50 participant<br>600 trial	48 participant<br>600 trial
Observations	205408	180427
Marginal R^2 / Conditional R^2	0.013 / 0.819	0.008 / 0.193

For Review Only

Velocity gain [%]			Catch-up saccades [Hz]			Anticipatory saccade	
Estimates	*SE*	*t*	Estimates	*SE*	*t*	Estimates	*SE*
92.70	1.16	79.67	0.33	0.04	9.26	0.36	0.04
0.76	0.80	0.94	-0.02	0.02	-0.72	0.04	0.02
-0.28	0.67	-0.42	0.00	0.02	-0.08	-0.03	0.02
-0.61	0.75	-0.81	-0.01	0.02	-0.59	-0.04	0.02
0.93	0.38	2.49	0.01	0.02	0.83	0.01	0.02
0.91	0.37	2.44	0.00	0.02	0.16	0.02	0.02
1.31	0.37	3.50	0.00	0.02	0.07	0.01	0.02
0.94	0.37	2.52	-0.01	0.02	-0.36	0.02	0.02
-0.12	0.37	-0.32	0.01	0.02	0.53	0.04	0.02
0.07	0.38	0.17	0.00	0.02	-0.03	0.00	0.02
-0.86	0.56	-1.54	0.00	0.01	-0.15	-0.01	0.01
-2.67	0.81	-3.31	0.05	0.03	1.86	-0.01	0.03
-2.60	0.94	-2.76	0.01	0.03	0.25	-0.02	0.03
0.06	0.53	0.11	-0.02	0.02	-0.89	-0.04	0.02
-0.23	0.53	-0.44	0.02	0.02	0.93	-0.03	0.02
-0.28	0.53	-0.53	0.01	0.02	0.39	-0.03	0.02
-0.43	0.53	-0.81	0.02	0.02	0.94	-0.03	0.02
0.23	0.53	0.43	0.00	0.02	-0.13	-0.03	0.02
-0.09	0.53	-0.17	0.00	0.02	0.01	-0.03	0.02
-2.70	0.53	-5.06	0.07	0.02	2.77	0.03	0.02
-2.34	0.53	-4.38	0.06	0.02	2.66	0.01	0.02
-1.82	0.53	-3.43	0.13	0.02	5.63	0.03	0.02
-2.69	0.53	-5.08	0.10	0.02	4.29	-0.01	0.02
-0.70	0.53	-1.33	0.11	0.02	4.42	0.06	0.02
-1.82	0.53	-3.42	0.14	0.02	5.84	0.03	0.02
-0.13	0.53	-0.25	0.07	0.02	2.88	0.04	0.02
0.12	0.53	0.23	0.13	0.02	5.30	0.00	0.02
1.36	0.53	2.58	0.03	0.02	1.24	0.02	0.02
1.25	0.53	2.35	0.08	0.02	3.15	-0.01	0.02
0.56	0.53	1.05	0.00	0.02	0.07	0.06	0.02
0.41	0.53	0.77	0.06	0.02	2.47	0.02	0.02
-2.20	0.75	-2.92	0.07	0.03	1.92	0.11	0.03
-2.46	0.75	-3.28	0.07	0.03	2.10	0.13	0.03
-0.10	0.75	-0.14	-0.01	0.03	-0.38	0.03	0.03
0.36	0.75	0.49	0.06	0.03	1.80	0.04	0.03
0.69	0.75	0.92	-0.02	0.03	-0.50	-0.01	0.03
1.11	0.75	1.48	0.00	0.03	-0.06	0.02	0.03
1.22	0.75	1.63	-0.05	0.03	-1.47	0.02	0.03
0.60	0.75	0.80	-0.06	0.03	-1.70	0.04	0.03
0.31	0.74	0.41	-0.02	0.03	-0.72	0.03	0.03
-0.27	0.75	-0.36	0.00	0.03	0.01	0.07	0.03
0.58	0.75	0.77	0.01	0.03	0.36	0.00	0.03
0.55	0.75	0.74	-0.02	0.03	-0.68	0.03	0.03
305.84			0.64			0.64	

For Review Only

2.67 trial<br>56.57 participant	0.00 trial<br>0.05 participant	0.00 trial<br>0.06 participant
21.64 participant.taskvs<br>12.15 participant.loadeasy<br>17.81 participant.loadhard<br>12.87 participant.taskvs:loadeasy<br>24.29 participant.taskvs:loadhard	0.01 participant.taskvs<br>0.00 participant.loadeasy<br>0.01 participant.loadhard<br>0.01 participant.taskvs:loadeasy<br>0.01 participant.taskvs:loadhard	0.01 participant.taskvs<br>0.01 participant.loadeasy<br>0.01 participant.loadhard<br>0.01 participant.taskvs:loadeasy<br>0.01 participant.taskvs:loadhard
-0.38 participant.taskvs<br>-0.13 participant.loadeasy<br>-0.04 participant.loadhard<br>0.46 participant.taskvs:loadeasy<br>0.30 participant.taskvs:loadhard	-0.22 participant.taskvs<br>0.22 participant.loadeasy<br>0.02 participant.loadhard<br>0.06 participant.taskvs:loadeasy<br>-0.03 participant.taskvs:loadhard	-0.38 participant.taskvs<br>0.05 participant.loadeasy<br>-0.30 participant.loadhard<br>0.11 participant.taskvs:loadeasy<br>0.24 participant.taskvs:loadhard
0.19	0.09	0.09
48 participant<br>600 trial	50 participant<br>600 trial	50 participant<br>600 trial
180427	185713	185713
0.010 / 0.193	0.005 / 0.092	0.001 / 0.089

For Review Only

es [Hz]
t
9.47
1.79
-1.38
-1.66
0.60
1.31
0.86
1.38
2.41
0.15
-1.01
-0.31
-0.88
-1.89
-1.29
-1.32
-1.21
-1.35
-1.19
1.43
0.55
1.12
-0.27
2.67
1.27
1.78
0.21
0.66
-0.49
2.33
0.86
3.32
3.86
0.98
1.29
-0.25
0.53
0.64
1.05
1.03
2.07
0.10
0.94

For Review Only

For Review Only

Eye.Parameter	Contrast	Time	Task	Estimate	*SE*	*df*
Pupil diameter	Single - Easy	0-0.5	Arithmetic	-0.08	0.03	112.31
Pupil diameter	Single - Hard	0-0.5	Arithmetic	-0.17	0.03	81.52
Pupil diameter	Easy - Hard	0-0.5	Arithmetic	-0.09	0.02	120.61
Pupil diameter	Single - Easy	0.5-1	Arithmetic	-0.09	0.03	112.32
Pupil diameter	Single - Hard	0.5-1	Arithmetic	-0.18	0.03	81.52
Pupil diameter	Easy - Hard	0.5-1	Arithmetic	-0.09	0.02	120.62
Pupil diameter	Single - Easy	1-1.5	Arithmetic	-0.13	0.03	112.35
Pupil diameter	Single - Hard	1-1.5	Arithmetic	-0.25	0.03	81.56
Pupil diameter	Easy - Hard	1-1.5	Arithmetic	-0.11	0.02	120.69
Pupil diameter	Single - Easy	1.5-2	Arithmetic	-0.13	0.03	112.31
Pupil diameter	Single - Hard	1.5-2	Arithmetic	-0.28	0.03	81.56
Pupil diameter	Easy - Hard	1.5-2	Arithmetic	-0.15	0.02	120.69
Pupil diameter	Single - Easy	2-2.5	Arithmetic	-0.09	0.03	112.34
Pupil diameter	Single - Hard	2-2.5	Arithmetic	-0.27	0.03	81.56
Pupil diameter	Easy - Hard	2-2.5	Arithmetic	-0.18	0.02	120.71
Pupil diameter	Single - Easy	2.5-3	Arithmetic	-0.07	0.03	112.34
Pupil diameter	Single - Hard	2.5-3	Arithmetic	-0.23	0.03	81.55
Pupil diameter	Easy - Hard	2.5-3	Arithmetic	-0.16	0.02	120.61
Pupil diameter	Single - Easy	3-3.5	Arithmetic	-0.07	0.03	113.67
Pupil diameter	Single - Hard	3-3.5	Arithmetic	-0.21	0.03	82
Pupil diameter	Easy - Hard	3-3.5	Arithmetic	-0.14	0.02	122.22
Pupil diameter	Single - Easy	0-0.5	Visuospatial	-0.09	0.03	108.26
Pupil diameter	Single - Hard	0-0.5	Visuospatial	-0.19	0.04	71.14
Pupil diameter	Easy - Hard	0-0.5	Visuospatial	-0.09	0.03	109.86
Pupil diameter	Single - Easy	0.5-1	Visuospatial	-0.12	0.03	108.26
Pupil diameter	Single - Hard	0.5-1	Visuospatial	-0.21	0.04	71.14
Pupil diameter	Easy - Hard	0.5-1	Visuospatial	-0.09	0.03	109.91
Pupil diameter	Single - Easy	1-1.5	Visuospatial	-0.16	0.03	108.27
Pupil diameter	Single - Hard	1-1.5	Visuospatial	-0.27	0.04	71.15
Pupil diameter	Easy - Hard	1-1.5	Visuospatial	-0.11	0.03	109.95
Pupil diameter	Single - Easy	1.5-2	Visuospatial	-0.15	0.03	108.24
Pupil diameter	Single - Hard	1.5-2	Visuospatial	-0.27	0.04	71.14
Pupil diameter	Easy - Hard	1.5-2	Visuospatial	-0.12	0.03	109.92
Pupil diameter	Single - Easy	2-2.5	Visuospatial	-0.12	0.03	108.29
Pupil diameter	Single - Hard	2-2.5	Visuospatial	-0.24	0.04	71.16
Pupil diameter	Easy - Hard	2-2.5	Visuospatial	-0.13	0.03	109.92
Pupil diameter	Single - Easy	2.5-3	Visuospatial	-0.09	0.03	108.28
Pupil diameter	Single - Hard	2.5-3	Visuospatial	-0.21	0.04	71.14
Pupil diameter	Easy - Hard	2.5-3	Visuospatial	-0.12	0.03	109.88
Pupil diameter	Single - Easy	3-3.5	Visuospatial	-0.08	0.03	109.09
Pupil diameter	Single - Hard	3-3.5	Visuospatial	-0.2	0.04	71.3
Pupil diameter	Easy - Hard	3-3.5	Visuospatial	-0.12	0.03	110.73
Gaze-to-target distance	Single - Easy	0-0.5	Arithmetic	0.03	0.02	177.06
Gaze-to-target distance	Single - Hard	0-0.5	Arithmetic	0	0.02	107.34
Gaze-to-target distance	Easy - Hard	0-0.5	Arithmetic	-0.02	0.02	117.07
Gaze-to-target distance	Single - Easy	0.5-1	Arithmetic	0	0.02	186.44
Gaze-to-target distance	Single - Hard	0.5-1	Arithmetic	-0.04	0.03	110.18
Gaze-to-target distance	Easy - Hard	0.5-1	Arithmetic	-0.04	0.02	122.99
Gaze-to-target distance	Single - Easy	1-1.5	Arithmetic	-0.02	0.02	182.98

For Review Only

Gaze-to-target distance	Single - Hard	1-1.5	Arithmetic	-0.08	0.03	108.81
Gaze-to-target distance	Easy - Hard	1-1.5	Arithmetic	-0.06	0.02	121.82
Gaze-to-target distance	Single - Easy	1.5-2	Arithmetic	-0.03	0.02	181.77
Gaze-to-target distance	Single - Hard	1.5-2	Arithmetic	-0.11	0.03	109.58
Gaze-to-target distance	Easy - Hard	1.5-2	Arithmetic	-0.08	0.02	121.42
Gaze-to-target distance	Single - Easy	2-2.5	Arithmetic	0	0.02	181.41
Gaze-to-target distance	Single - Hard	2-2.5	Arithmetic	-0.06	0.03	109.78
Gaze-to-target distance	Easy - Hard	2-2.5	Arithmetic	-0.05	0.02	121.16
Gaze-to-target distance	Single - Easy	2.5-3	Arithmetic	0.01	0.02	180.12
Gaze-to-target distance	Single - Hard	2.5-3	Arithmetic	-0.04	0.03	109.24
Gaze-to-target distance	Easy - Hard	2.5-3	Arithmetic	-0.04	0.02	119.98
Gaze-to-target distance	Single - Easy	3-3.5	Arithmetic	0.01	0.02	187.51
Gaze-to-target distance	Single - Hard	3-3.5	Arithmetic	-0.01	0.03	111.13
Gaze-to-target distance	Easy - Hard	3-3.5	Arithmetic	-0.02	0.02	123.11
Gaze-to-target distance	Single - Easy	0-0.5	Visuospatial	-0.08	0.03	76.07
Gaze-to-target distance	Single - Hard	0-0.5	Visuospatial	-0.06	0.04	65.32
Gaze-to-target distance	Easy - Hard	0-0.5	Visuospatial	0.03	0.03	97.89
Gaze-to-target distance	Single - Easy	0.5-1	Visuospatial	-0.16	0.03	77.47
Gaze-to-target distance	Single - Hard	0.5-1	Visuospatial	-0.14	0.04	66.07
Gaze-to-target distance	Easy - Hard	0.5-1	Visuospatial	0.02	0.03	101.94
Gaze-to-target distance	Single - Easy	1-1.5	Visuospatial	-0.19	0.03	77.07
Gaze-to-target distance	Single - Hard	1-1.5	Visuospatial	-0.17	0.04	66.04
Gaze-to-target distance	Easy - Hard	1-1.5	Visuospatial	0.02	0.03	101.31
Gaze-to-target distance	Single - Easy	1.5-2	Visuospatial	-0.15	0.03	76.56
Gaze-to-target distance	Single - Hard	1.5-2	Visuospatial	-0.16	0.04	65.67
Gaze-to-target distance	Easy - Hard	1.5-2	Visuospatial	-0.01	0.03	99.96
Gaze-to-target distance	Single - Easy	2-2.5	Visuospatial	-0.09	0.03	76.52
Gaze-to-target distance	Single - Hard	2-2.5	Visuospatial	-0.1	0.04	65.66
Gaze-to-target distance	Easy - Hard	2-2.5	Visuospatial	-0.01	0.03	99.23
Gaze-to-target distance	Single - Easy	2.5-3	Visuospatial	-0.07	0.03	76.37
Gaze-to-target distance	Single - Hard	2.5-3	Visuospatial	-0.07	0.04	65.62
Gaze-to-target distance	Easy - Hard	2.5-3	Visuospatial	0.01	0.03	98.96
Gaze-to-target distance	Single - Easy	3-3.5	Visuospatial	-0.08	0.03	77.22
Gaze-to-target distance	Single - Hard	3-3.5	Visuospatial	-0.05	0.04	65.93
Gaze-to-target distance	Easy - Hard	3-3.5	Visuospatial	0.03	0.03	100.45
Velocity gain	Single - Easy	0-0.5	Arithmetic	0.28	0.67	122.24
Velocity gain	Single - Hard	0-0.5	Arithmetic	0.61	0.75	96.57
Velocity gain	Easy - Hard	0-0.5	Arithmetic	0.33	0.56	221.29
Velocity gain	Single - Easy	0.5-1	Arithmetic	2.98	0.67	127.6
Velocity gain	Single - Hard	0.5-1	Arithmetic	2.95	0.76	99.01
Velocity gain	Easy - Hard	0.5-1	Arithmetic	-0.03	0.57	238.66
Velocity gain	Single - Easy	1-1.5	Arithmetic	2.1	0.67	125.62
Velocity gain	Single - Hard	1-1.5	Arithmetic	3.3	0.75	97.83
Velocity gain	Easy - Hard	1-1.5	Arithmetic	1.2	0.57	235.43
Velocity gain	Single - Easy	1.5-2	Arithmetic	0.98	0.67	124.94
Velocity gain	Single - Hard	1.5-2	Arithmetic	2.43	0.75	98.49
Velocity gain	Easy - Hard	1.5-2	Arithmetic	1.45	0.57	234.21
Velocity gain	Single - Easy	2-2.5	Arithmetic	0.41	0.67	124.73
Velocity gain	Single - Hard	2-2.5	Arithmetic	0.49	0.75	98.66
Velocity gain	Easy - Hard	2-2.5	Arithmetic	0.08	0.57	233.45

For Review Only

Velocity gain	Single - Easy	2.5-3	Arithmetic	-1.08	0.67	124
Velocity gain	Single - Hard	2.5-3	Arithmetic	-0.64	0.75	98.2
Velocity gain	Easy - Hard	2.5-3	Arithmetic	0.45	0.56	229.93
Velocity gain	Single - Easy	3-3.5	Arithmetic	-0.29	0.67	128.18
Velocity gain	Single - Hard	3-3.5	Arithmetic	0.2	0.76	99.81
Velocity gain	Easy - Hard	3-3.5	Arithmetic	0.49	0.57	239.31
Velocity gain	Single - Easy	0-0.5	Visuospatial	2.95	0.82	84.35
Velocity gain	Single - Hard	0-0.5	Visuospatial	3.21	0.95	71.36
Velocity gain	Easy - Hard	0-0.5	Visuospatial	0.26	0.57	196.31
Velocity gain	Single - Easy	0.5-1	Visuospatial	7.84	0.82	86.31
Velocity gain	Single - Hard	0.5-1	Visuospatial	8.01	0.95	72.45
Velocity gain	Easy - Hard	0.5-1	Visuospatial	0.17	0.59	210.68
Velocity gain	Single - Easy	1-1.5	Visuospatial	4.87	0.82	85.75
Velocity gain	Single - Hard	1-1.5	Visuospatial	5.53	0.95	72.41
Velocity gain	Easy - Hard	1-1.5	Visuospatial	0.67	0.58	208.51
Velocity gain	Single - Easy	1.5-2	Visuospatial	2.96	0.82	85.04
Velocity gain	Single - Hard	1.5-2	Visuospatial	3.92	0.95	71.87
Velocity gain	Easy - Hard	1.5-2	Visuospatial	0.96	0.58	203.7
Velocity gain	Single - Easy	2-2.5	Visuospatial	1.86	0.82	84.98
Velocity gain	Single - Hard	2-2.5	Visuospatial	2.49	0.95	71.86
Velocity gain	Easy - Hard	2-2.5	Visuospatial	0.63	0.58	201.11
Velocity gain	Single - Easy	2.5-3	Visuospatial	1.28	0.82	84.77
Velocity gain	Single - Hard	2.5-3	Visuospatial	2.23	0.95	71.8
Velocity gain	Easy - Hard	2.5-3	Visuospatial	0.95	0.58	200.11
Velocity gain	Single - Easy	3-3.5	Visuospatial	1.8	0.82	85.95
Velocity gain	Single - Hard	3-3.5	Visuospatial	2.24	0.95	72.24
Velocity gain	Easy - Hard	3-3.5	Visuospatial	0.44	0.58	205.44
Catch-up saccades	Single - Easy	0-0.5	Arithmetic	0	0.02	448.4
Catch-up saccades	Single - Hard	0-0.5	Arithmetic	0.01	0.02	188.13
Catch-up saccades	Easy - Hard	0-0.5	Arithmetic	0.01	0.02	321.23
Catch-up saccades	Single - Easy	0.5-1	Arithmetic	-0.07	0.02	490
Catch-up saccades	Single - Hard	0.5-1	Arithmetic	-0.05	0.02	197.85
Catch-up saccades	Easy - Hard	0.5-1	Arithmetic	0.01	0.02	356.58
Catch-up saccades	Single - Easy	1-1.5	Arithmetic	-0.13	0.02	477.47
Catch-up saccades	Single - Hard	1-1.5	Arithmetic	-0.09	0.02	194.17
Catch-up saccades	Easy - Hard	1-1.5	Arithmetic	0.04	0.02	350.4
Catch-up saccades	Single - Easy	1.5-2	Arithmetic	-0.1	0.02	468.53
Catch-up saccades	Single - Hard	1.5-2	Arithmetic	-0.13	0.02	195.42
Catch-up saccades	Easy - Hard	1.5-2	Arithmetic	-0.02	0.02	345
Catch-up saccades	Single - Easy	2-2.5	Arithmetic	-0.07	0.02	468.27
Catch-up saccades	Single - Hard	2-2.5	Arithmetic	-0.11	0.02	196.2
Catch-up saccades	Easy - Hard	2-2.5	Arithmetic	-0.05	0.02	344.08
Catch-up saccades	Single - Easy	2.5-3	Arithmetic	-0.03	0.02	463.15
Catch-up saccades	Single - Hard	2.5-3	Arithmetic	-0.06	0.02	193.89
Catch-up saccades	Easy - Hard	2.5-3	Arithmetic	-0.03	0.02	338.02
Catch-up saccades	Single - Easy	3-3.5	Arithmetic	0	0.02	496.21
Catch-up saccades	Single - Hard	3-3.5	Arithmetic	-0.05	0.02	200.87
Catch-up saccades	Easy - Hard	3-3.5	Arithmetic	-0.05	0.02	357.19
Catch-up saccades	Single - Easy	0-0.5	Visuospatial	-0.05	0.02	237.74
Catch-up saccades	Single - Hard	0-0.5	Visuospatial	0.01	0.02	244.13

For Review Only

Catch-up saccades	Easy - Hard	0-0.5	Visuospatial	0.05	0.02	274.05
Catch-up saccades	Single - Easy	0.5-1	Visuospatial	-0.18	0.02	252.53
Catch-up saccades	Single - Hard	0.5-1	Visuospatial	-0.13	0.02	256.55
Catch-up saccades	Easy - Hard	0.5-1	Visuospatial	0.05	0.02	302.07
Catch-up saccades	Single - Easy	1-1.5	Visuospatial	-0.17	0.02	247.84
Catch-up saccades	Single - Hard	1-1.5	Visuospatial	-0.16	0.02	256.23
Catch-up saccades	Easy - Hard	1-1.5	Visuospatial	0.01	0.02	296.88
Catch-up saccades	Single - Easy	1.5-2	Visuospatial	-0.14	0.02	242.33
Catch-up saccades	Single - Hard	1.5-2	Visuospatial	-0.13	0.02	250.09
Catch-up saccades	Easy - Hard	1.5-2	Visuospatial	0.01	0.02	287.99
Catch-up saccades	Single - Easy	2-2.5	Visuospatial	-0.07	0.02	241.96
Catch-up saccades	Single - Hard	2-2.5	Visuospatial	-0.06	0.02	248.95
Catch-up saccades	Easy - Hard	2-2.5	Visuospatial	0	0.02	282.93
Catch-up saccades	Single - Easy	2.5-3	Visuospatial	-0.05	0.02	241.27
Catch-up saccades	Single - Hard	2.5-3	Visuospatial	-0.07	0.02	249
Catch-up saccades	Easy - Hard	2.5-3	Visuospatial	-0.02	0.02	282.17
Catch-up saccades	Single - Easy	3-3.5	Visuospatial	-0.06	0.02	250.03
Catch-up saccades	Single - Hard	3-3.5	Visuospatial	-0.03	0.02	254.29
Catch-up saccades	Easy - Hard	3-3.5	Visuospatial	0.03	0.02	292.07
Anticipatory saccades	Single - Easy	0-0.5	Arithmetic	0.03	0.02	321.08
Anticipatory saccades	Single - Hard	0-0.5	Arithmetic	0.04	0.02	183.16
Anticipatory saccades	Easy - Hard	0-0.5	Arithmetic	0.01	0.02	256.62
Anticipatory saccades	Single - Easy	0.5-1	Arithmetic	-0.01	0.02	350.06
Anticipatory saccades	Single - Hard	0.5-1	Arithmetic	0.02	0.02	192.9
Anticipatory saccades	Easy - Hard	0.5-1	Arithmetic	0.03	0.02	284.53
Anticipatory saccades	Single - Easy	1-1.5	Arithmetic	0	0.02	341.18
Anticipatory saccades	Single - Hard	1-1.5	Arithmetic	0.04	0.02	189.22
Anticipatory saccades	Easy - Hard	1-1.5	Arithmetic	0.04	0.02	279.49
Anticipatory saccades	Single - Easy	1.5-2	Arithmetic	-0.04	0.02	335.03
Anticipatory saccades	Single - Hard	1.5-2	Arithmetic	0.01	0.02	190.46
Anticipatory saccades	Easy - Hard	1.5-2	Arithmetic	0.04	0.02	275.25
Anticipatory saccades	Single - Easy	2-2.5	Arithmetic	-0.01	0.02	334.79
Anticipatory saccades	Single - Hard	2-2.5	Arithmetic	0.03	0.02	191.24
Anticipatory saccades	Easy - Hard	2-2.5	Arithmetic	0.05	0.02	274.5
Anticipatory saccades	Single - Easy	2.5-3	Arithmetic	0.01	0.02	331.31
Anticipatory saccades	Single - Hard	2.5-3	Arithmetic	0.05	0.02	188.93
Anticipatory saccades	Easy - Hard	2.5-3	Arithmetic	0.04	0.02	269.76
Anticipatory saccades	Single - Easy	3-3.5	Arithmetic	-0.03	0.02	353.09
Anticipatory saccades	Single - Hard	3-3.5	Arithmetic	0.02	0.02	195.7
Anticipatory saccades	Easy - Hard	3-3.5	Arithmetic	0.05	0.02	284.12
Anticipatory saccades	Single - Easy	0-0.5	Visuospatial	0.04	0.02	168.59
Anticipatory saccades	Single - Hard	0-0.5	Visuospatial	0.06	0.02	145.31
Anticipatory saccades	Easy - Hard	0-0.5	Visuospatial	0.03	0.02	656.37
Anticipatory saccades	Single - Easy	0.5-1	Visuospatial	-0.11	0.02	177.76
Anticipatory saccades	Single - Hard	0.5-1	Visuospatial	-0.08	0.02	151.16
Anticipatory saccades	Easy - Hard	0.5-1	Visuospatial	0.03	0.02	738.27
Anticipatory saccades	Single - Easy	1-1.5	Visuospatial	-0.02	0.02	174.85
Anticipatory saccades	Single - Hard	1-1.5	Visuospatial	0.02	0.02	150.98
Anticipatory saccades	Easy - Hard	1-1.5	Visuospatial	0.05	0.02	724.98
Anticipatory saccades	Single - Easy	1.5-2	Visuospatial	-0.02	0.02	171.44

For Review Only

Anticipatory saccades	Single - Hard	1.5-2	Visuospatial	0.01	0.02	148.09
Anticipatory saccades	Easy - Hard	1.5-2	Visuospatial	0.03	0.02	699.16
Anticipatory saccades	Single - Easy	2-2.5	Visuospatial	-0.03	0.02	171.21
Anticipatory saccades	Single - Hard	2-2.5	Visuospatial	0.02	0.02	147.56
Anticipatory saccades	Easy - Hard	2-2.5	Visuospatial	0.05	0.02	683.76
Anticipatory saccades	Single - Easy	2.5-3	Visuospatial	-0.01	0.02	170.78
Anticipatory saccades	Single - Hard	2.5-3	Visuospatial	0	0.02	147.58
Anticipatory saccades	Easy - Hard	2.5-3	Visuospatial	0.02	0.02	681.22
Anticipatory saccades	Single - Easy	3-3.5	Visuospatial	-0.02	0.02	176.05
Anticipatory saccades	Single - Hard	3-3.5	Visuospatial	0.01	0.02	149.98
Anticipatory saccades	Easy - Hard	3-3.5	Visuospatial	0.03	0.02	711.36

For Review Only

t	*p*	Lower.CI	Upper.CI	Effect.Size	BF10
-2.97	.011	-0.14	-0.01	-0.08	51.17
-5.32	< .001	-0.25	-0.09	-0.17	86612.88
-3.71	< .001	-0.15	-0.03	-0.09	671.89
-3.57	.002	-0.15	-0.03	-0.09	387.56
-5.58	< .001	-0.25	-0.1	-0.18	> 100,000
-3.43	.002	-0.15	-0.02	-0.09	381.62
-5.24	< .001	-0.2	-0.07	-0.14	87991.86
-7.78	< .001	-0.32	-0.17	-0.25	> 100,000
-4.51	< .001	-0.17	-0.05	-0.11	59664.62
-5.12	< .001	-0.19	-0.07	-0.13	56131.36
-8.9	< .001	-0.36	-0.2	-0.29	> 100,000
-6.05	< .001	-0.21	-0.09	-0.15	> 100,000
-3.49	.002	-0.15	-0.03	-0.09	229.18
-8.41	< .001	-0.34	-0.19	-0.27	> 100,000
-7.12	< .001	-0.24	-0.12	-0.18	> 100,000
-2.67	.026	-0.13	-0.01	-0.07	14.72
-7.31	< .001	-0.31	-0.15	-0.24	> 100,000
-6.57	< .001	-0.22	-0.1	-0.17	> 100,000
-2.77	.020	-0.13	-0.01	-0.07	32.03
-6.6	< .001	-0.29	-0.13	-0.21	> 100,000
-5.55	< .001	-0.2	-0.08	-0.14	> 100,000
-3.54	.002	-0.16	-0.03	-0.09	485.78
-5.08	< .001	-0.28	-0.1	-0.19	15621.98
-3.56	.002	-0.16	-0.03	-0.09	237.47
-4.39	< .001	-0.18	-0.05	-0.12	40491.22
-5.72	< .001	-0.3	-0.12	-0.21	> 100,000
-3.61	.001	-0.16	-0.03	-0.1	313.9
-6.21	< .001	-0.23	-0.1	-0.17	> 100,000
-7.38	< .001	-0.36	-0.18	-0.27	> 100,000
-4.1	< .001	-0.17	-0.04	-0.11	3001.23
-5.66	< .001	-0.21	-0.08	-0.15	> 100,000
-7.39	< .001	-0.36	-0.18	-0.27	> 100,000
-4.68	< .001	-0.18	-0.06	-0.12	93842.76
-4.4	< .001	-0.18	-0.05	-0.12	2907.57
-6.6	< .001	-0.33	-0.15	-0.25	> 100,000
-4.83	< .001	-0.19	-0.06	-0.13	52212.48
-3.55	.002	-0.16	-0.03	-0.09	184.14
-5.85	< .001	-0.3	-0.12	-0.22	> 100,000
-4.64	< .001	-0.18	-0.06	-0.12	8431.03
-3.18	.006	-0.15	-0.02	-0.09	94.35
-5.48	< .001	-0.29	-0.11	-0.2	34207.65
-4.47	< .001	-0.18	-0.05	-0.12	2455.07
1.22	.671	-0.02	0.08	0.03	0.5
0.15	1	-0.06	0.06	0	0.16
-0.9	1	-0.08	0.04	-0.03	0.44
0.07	1	-0.05	0.05	0	0.17
-1.43	.467	-0.1	0.03	-0.05	1.02
-1.55	.374	-0.1	0.02	-0.05	0.79
-0.98	.988	-0.07	0.03	-0.03	0.6

For Review Only

-3.25	.005	-0.14	-0.02	-0.11	5.26
-2.52	.039	-0.12	0	-0.08	2.98
-1.41	.48	-0.08	0.02	-0.04	0.37
-4.53	< .001	-0.17	-0.05	-0.15	32.53
-3.5	0.002	-0.14	-0.03	-0.11	7.33
-0.22	1	-0.05	0.05	-0.01	0.17
-2.3	0.07	-0.12	0	-0.08	4.57
-2.2	0.088	-0.11	0.01	-0.07	3.45
0.25	1	-0.04	0.06	0.01	0.16
-1.48	0.426	-0.1	0.02	-0.05	1.04
-1.76	0.243	-0.1	0.02	-0.06	1.53
0.28	1	-0.04	0.06	0.01	0.16
-0.43	1	-0.07	0.05	-0.01	0.23
-0.69	1	-0.08	0.04	-0.02	0.27
-2.63	0.031	-0.16	-0.01	-0.11	10.3
-1.52	0.403	-0.15	0.03	-0.07	1.05
0.99	0.97	-0.04	0.09	0.03	0.25
-5.23	< .001	-0.24	-0.09	-0.22	2903.85
-3.83	< .001	-0.23	-0.05	-0.19	86.3
0.82	1	-0.04	0.09	0.03	0.17
-5.92	< .001	-0.26	-0.11	-0.25	1091.13
-4.45	< .001	-0.26	-0.07	-0.22	248.35
0.76	1	-0.04	0.08	0.03	0.16
-4.88	< .001	-0.23	-0.08	-0.2	423.56
-4.29	< .001	-0.25	-0.07	-0.21	181.25
-0.25	1	-0.07	0.06	-0.01	0.21
-3	0.011	-0.17	-0.02	-0.12	9.25
-2.81	0.02	-0.2	-0.01	-0.14	4.98
-0.4	1	-0.07	0.05	-0.01	0.17
-2.36	0.063	-0.15	0	-0.1	4.08
-1.81	0.224	-0.16	0.02	-0.09	1.22
0.24	1	-0.06	0.07	0.01	0.16
-2.43	0.052	-0.15	0	-0.1	6.3
-1.31	0.581	-0.14	0.04	-0.06	0.46
1.04	0.9	-0.04	0.09	0.04	0.25
0.42	1	-1.34	1.89	0.01	0.17
0.81	1	-1.22	2.44	0.03	0.32
0.6	1	-1.01	1.68	0.02	0.27
4.42	< .001	1.34	4.61	0.14	207.94
3.9	< .001	1.11	4.79	0.14	49.89
-0.05	1	-1.4	1.34	0	0.16
3.12	0.007	0.47	3.72	0.1	21.96
4.38	< .001	1.47	5.13	0.15	23.33
2.12	0.104	-0.16	2.57	0.06	0.84
1.46	0.439	-0.65	2.61	0.05	0.6
3.22	0.005	0.59	4.26	0.11	4.94
2.55	0.034	0.08	2.81	0.07	0.74
0.61	1	-1.22	2.03	0.02	0.18
0.65	1	-1.35	2.33	0.02	0.18
0.15	1	-1.28	1.45	0	0.16

For Review Only

-1.62	0.323	-2.71	0.54	-0.05	0.39
-0.84	1	-2.47	1.2	-0.03	0.24
0.79	1	-0.91	1.81	0.02	0.16
-0.42	1	-1.92	1.35	-0.01	0.17
0.26	1	-1.64	2.04	0.01	0.16
0.85	1	-0.89	1.86	0.02	0.21
3.6	0.002	0.95	4.94	0.14	808.85
3.39	0.003	0.89	5.53	0.15	89.15
0.46	1	-1.12	1.65	0.01	0.16
9.54	< .001	5.84	9.85	0.37	> 100,000
8.43	< .001	5.68	10.34	0.37	> 100,000
0.28	1	-1.25	1.58	0.01	0.17
5.93	< .001	2.86	6.87	0.23	37021.66
5.82	< .001	3.2	7.86	0.26	2962.99
1.14	0.764	-0.74	2.08	0.03	0.25
3.61	0.002	0.96	4.96	0.14	134.2
4.13	< .001	1.59	6.24	0.18	270.6
1.65	0.301	-0.44	2.36	0.04	0.68
2.27	0.077	-0.14	3.86	0.09	2.33
2.63	0.032	0.17	4.82	0.12	12.08
1.09	0.826	-0.76	2.03	0.03	0.5
1.56	0.366	-0.72	3.28	0.06	1.21
2.35	0.065	-0.1	4.55	0.1	5.24
1.64	0.306	-0.45	2.34	0.04	0.68
2.2	0.092	-0.2	3.81	0.08	7.37
2.36	0.063	-0.08	4.57	0.1	3.52
0.75	1	-0.96	1.84	0.02	0.16
0.08	1	-0.05	0.05	0	0.16
0.59	1	-0.04	0.07	0.02	0.23
0.59	1	-0.04	0.06	0.01	0.22
-3.23	0.004	-0.11	-0.02	-0.08	1.18
-2.15	0.099	-0.11	0.01	-0.06	0.56
0.7	1	-0.04	0.07	0.02	0.17
-6.66	< .001	-0.18	-0.09	-0.16	650.91
-3.82	< .001	-0.15	-0.03	-0.1	8.58
2.1	0.11	-0.01	0.09	0.05	0.64
-5.22	< .001	-0.15	-0.06	-0.12	800.06
-5.42	< .001	-0.18	-0.07	-0.15	297
-1.07	0.853	-0.07	0.03	-0.03	0.18
-3.37	0.002	-0.12	-0.02	-0.08	12.56
-4.85	< .001	-0.17	-0.06	-0.13	2509.34
-2.21	0.084	-0.1	0	-0.05	0.83
-1.41	0.48	-0.08	0.02	-0.03	0.43
-2.65	0.026	-0.12	-0.01	-0.07	5.71
-1.62	0.32	-0.08	0.02	-0.04	0.62
-0.01	1	-0.05	0.05	0	0.16
-1.96	0.155	-0.1	0.01	-0.05	1
-2.17	0.093	-0.1	0.01	-0.05	3.17
-2.19	0.089	-0.1	0	-0.06	1.9
0.31	1	-0.05	0.06	0.01	0.17

For Review Only

2.58	0.031	0	0.11	0.06	3.4
-8.09	< .001	-0.23	-0.13	-0.21	3673.32
-5.85	< .001	-0.18	-0.08	-0.15	12.49
2.38	0.054	0	0.1	0.06	1.07
-7.68	< .001	-0.22	-0.12	-0.2	> 100,000
-7.15	< .001	-0.21	-0.1	-0.18	37195.96
0.61	1	-0.04	0.06	0.02	0.17
-6.2	< .001	-0.19	-0.08	-0.16	5445.2
-6.02	< .001	-0.18	-0.08	-0.15	> 100,000
0.24	1	-0.05	0.06	0.01	0.16
-3.06	0.007	-0.12	-0.01	-0.08	17.19
-2.89	0.013	-0.12	-0.01	-0.07	2.68
0.2	1	-0.05	0.06	0	0.16
-2.42	0.049	-0.11	0	-0.06	7.93
-3.17	0.005	-0.12	-0.02	-0.08	64.8
-0.75	1	-0.07	0.04	-0.02	0.22
-2.78	0.017	-0.12	-0.01	-0.07	6.95
-1.37	0.518	-0.08	0.02	-0.04	0.39
1.48	0.42	-0.02	0.08	0.04	0.6
1.38	0.51	-0.02	0.08	0.03	0.37
1.66	0.298	-0.02	0.09	0.04	0.65
0.48	1	-0.04	0.06	0.01	0.18
-0.33	1	-0.06	0.04	-0.01	0.16
1.06	0.867	-0.03	0.08	0.03	0.25
1.46	0.437	-0.02	0.08	0.04	0.34
0.05	1	-0.05	0.05	0	0.16
1.92	0.169	-0.01	0.1	0.05	0.46
2.02	0.132	-0.01	0.09	0.05	0.36
-1.76	0.239	-0.08	0.01	-0.04	0.87
0.32	1	-0.05	0.06	0.01	0.16
2.03	0.129	-0.01	0.09	0.05	0.63
-0.72	1	-0.06	0.03	-0.02	0.22
1.42	0.471	-0.02	0.09	0.04	0.48
2.24	0.078	0	0.1	0.06	6.94
0.59	1	-0.04	0.06	0.01	0.18
2.16	0.097	-0.01	0.1	0.06	1.53
1.78	0.23	-0.01	0.09	0.04	1.09
-1.37	0.518	-0.08	0.02	-0.03	0.58
0.74	1	-0.04	0.07	0.02	0.21
2.12	0.105	-0.01	0.1	0.05	1.43
1.54	0.376	-0.02	0.09	0.04	0.68
2.54	0.037	0	0.12	0.07	3.26
1.41	0.475	-0.02	0.07	0.03	0.66
-4.7	< .001	-0.17	-0.05	-0.13	57.29
-3.32	0.003	-0.14	-0.02	-0.09	6.71
1.55	0.367	-0.02	0.07	0.03	0.29
-1.02	0.923	-0.08	0.03	-0.03	0.25
1.01	0.947	-0.03	0.08	0.03	0.25
2.59	0.029	0	0.09	0.06	3.16
-0.82	1	-0.08	0.04	-0.02	0.21

For Review Only

0.56	1	-0.05	0.07	0.02	0.17
1.76	0.239	-0.01	0.08	0.04	0.52
-1.2	0.699	-0.08	0.03	-0.03	0.41
0.88	1	-0.04	0.08	0.03	0.25
2.67	0.023	0	0.09	0.06	4.82
-0.61	1	-0.07	0.04	-0.02	0.17
0.17	1	-0.06	0.06	0	0.16
0.99	0.975	-0.03	0.06	0.02	0.23
-0.99	0.976	-0.08	0.03	-0.03	0.29
0.39	1	-0.05	0.07	0.01	0.16
1.75	0.242	-0.01	0.08	0.04	0.73

For Review Only

BF01
0.02
< 0.01
< 0.01
< 0.01
< 0.01
< 0.01
< 0.01
< 0.01
< 0.01
< 0.01
< 0.01
< 0.01
< 0.01
< 0.01
< 0.01
0.07
< 0.01
< 0.01
0.03
< 0.01
< 0.01
< 0.01
< 0.01
< 0.01
< 0.01
< 0.01
< 0.01
< 0.01
< 0.01
< 0.01
< 0.01
< 0.01
< 0.01
< 0.01
< 0.01
< 0.01
0.01
< 0.01
< 0.01
0.01
< 0.01
< 0.01
1.98
6.33
2.25
5.96
0.98
1.26
1.66

For Review Only

0.19
0.34
2.74
0.03
0.14
5.72
0.22
0.29
6.44
0.96
0.65
6.37
4.33
3.69
0.1
0.96
3.97
< .01
0.01
5.97
< .01
< .01
6.44
< .01
0.01
4.72
0.11
0.2
5.76
0.24
0.82
6.31
0.16
2.19
4.01
5.94
3.11
3.71
< .01
0.02
6.07
0.05
0.04
1.19
1.68
0.2
1.36
5.51
5.69
6.44

For Review Only

2.56
4.13
6.08
5.78
6.41
4.73
< .01
0.01
6.39
< .01
< .01
5.84
< .01
< .01
4.01
0.01
< .01
1.48
0.43
0.08
2
0.82
0.19
1.47
0.14
0.28
6.18
6.13
4.41
4.5
0.85
1.77
6.02
< .01
0.12
1.57
< .01
< .01
5.67
0.08
< .01
1.2
2.31
0.18
1.62
6.35
1
0.32
0.53
6

For Review Only

0.29
< .01
0.08
0.93
< .01
< .01
5.94
< .01
< .01
6.25
0.06
0.37
6.43
0.13
0.02
4.6
0.14
2.53
1.66
2.7
1.55
5.52
6.32
4.07
2.93
6.29
2.19
2.79
1.14
6.17
1.59
4.61
2.08
0.14
5.68
0.65
0.92
1.71
4.83
0.7
1.47
0.31
1.53
0.02
0.15
3.39
4.07
3.97
0.32
4.8

For Review Only

5.73
1.94
2.44
3.97
0.21
5.84
6.4
4.28
3.39
6.23
1.37

For Review Only

Eye.Parame	Workload c	Task condit	Time	Estimate	*SE*	*df*	*t*	*p*
Pupil diame	Single - Eas	Arithmetic	0-0.5	0.02	0.03	167.8	0.53	1
Pupil diame	Single - Har	Arithmetic	0-0.5	0.02	0.04	108.45	0.45	1
Pupil diame	Easy - Hard	Arithmetic	0-0.5	0	0.03	156.29	0.01	1
Pupil diame	Single - Eas	Arithmetic	0.5-1	0.02	0.03	167.81	0.74	1
Pupil diame	Single - Har	Arithmetic	0.5-1	0.03	0.04	108.46	0.85	1
Pupil diame	Easy - Hard	Arithmetic	0.5-1	0.01	0.03	156.36	0.27	1
Pupil diame	Single - Eas	Arithmetic	1-1.5	0.03	0.03	167.85	0.9	1
Pupil diame	Single - Har	Arithmetic	1-1.5	0.02	0.04	108.51	0.6	1
Pupil diame	Easy - Hard	Arithmetic	1-1.5	-0.01	0.03	156.45	-0.18	1
Pupil diame	Single - Eas	Arithmetic	1.5-2	0.02	0.03	167.77	0.53	1
Pupil diame	Single - Har	Arithmetic	1.5-2	-0.01	0.04	108.5	-0.34	1
Pupil diame	Easy - Hard	Arithmetic	1.5-2	-0.03	0.03	156.42	-0.91	1
Pupil diame	Single - Eas	Arithmetic	2-2.5	0.03	0.03	167.86	0.82	1
Pupil diame	Single - Har	Arithmetic	2-2.5	-0.03	0.04	108.51	-0.7	1
Pupil diame	Easy - Hard	Arithmetic	2-2.5	-0.05	0.03	156.43	-1.62	0.32
Pupil diame	Single - Eas	Arithmetic	2.5-3	0.02	0.03	167.84	0.78	1
Pupil diame	Single - Har	Arithmetic	2.5-3	-0.02	0.04	108.48	-0.5	1
Pupil diame	Easy - Hard	Arithmetic	2.5-3	-0.04	0.03	156.32	-1.35	0.54
Pupil diame	Single - Eas	Arithmetic	3-3.5	0.01	0.03	170.1	0.39	1
Pupil diame	Single - Har	Arithmetic	3-3.5	-0.01	0.04	109.16	-0.26	1
Pupil diame	Easy - Hard	Arithmetic	3-3.5	-0.02	0.03	158.4	-0.68	1
Gaze-to-tar	Single - Eas	Arithmetic	0-0.5	0.11	0.04	107.82	3.07	0.008
Gaze-to-tar	Single - Har	Arithmetic	0-0.5	0.06	0.04	97.01	1.62	0.327
Gaze-to-tar	Easy - Hard	Arithmetic	0-0.5	-0.05	0.03	158.37	-1.57	0.357
Gaze-to-tar	Single - Eas	Arithmetic	0.5-1	0.17	0.04	111.39	4.7	< .001
Gaze-to-tar	Single - Har	Arithmetic	0.5-1	0.11	0.04	99.29	2.87	0.015
Gaze-to-tar	Easy - Hard	Arithmetic	0.5-1	-0.06	0.03	168.2	-1.92	0.169
Gaze-to-tar	Single - Eas	Arithmetic	1-1.5	0.17	0.04	110.2	4.7	< .001
Gaze-to-tar	Single - Har	Arithmetic	1-1.5	0.08	0.04	98.68	2.27	0.076
Gaze-to-tar	Easy - Hard	Arithmetic	1-1.5	-0.08	0.03	166.49	-2.64	0.027
Gaze-to-tar	Single - Eas	Arithmetic	1.5-2	0.12	0.04	109.37	3.52	0.002
Gaze-to-tar	Single - Har	Arithmetic	1.5-2	0.05	0.04	98.45	1.23	0.664
Gaze-to-tar	Easy - Hard	Arithmetic	1.5-2	-0.08	0.03	164.52	-2.55	0.035
Gaze-to-tar	Single - Eas	Arithmetic	2-2.5	0.09	0.04	109.25	2.54	0.037
Gaze-to-tar	Single - Har	Arithmetic	2-2.5	0.05	0.04	98.51	1.25	0.638
Gaze-to-tar	Easy - Hard	Arithmetic	2-2.5	-0.04	0.03	163.42	-1.4	0.492
Gaze-to-tar	Single - Eas	Arithmetic	2.5-3	0.08	0.04	108.81	2.25	0.079
Gaze-to-tar	Single - Har	Arithmetic	2.5-3	0.03	0.04	98.23	0.81	1
Gaze-to-tar	Easy - Hard	Arithmetic	2.5-3	-0.05	0.03	162.1	-1.6	0.336
Gaze-to-tar	Single - Eas	Arithmetic	3-3.5	0.08	0.04	111.32	2.33	0.065
Gaze-to-tar	Single - Har	Arithmetic	3-3.5	0.04	0.04	99.46	1.02	0.933
Gaze-to-tar	Easy - Hard	Arithmetic	3-3.5	-0.04	0.03	166.55	-1.44	0.457
Velocity gai	Single - Eas	Arithmetic	0-0.5	-2.67	0.81	203.61	-3.31	0.003
Velocity gai	Single - Har	Arithmetic	0-0.5	-2.6	0.94	122.72	-2.76	0.02
Velocity gai	Easy - Hard	Arithmetic	0-0.5	0.07	0.73	341.36	0.1	1
Velocity gai	Single - Eas	Arithmetic	0.5-1	-4.87	0.82	214.45	-5.96	< .001
Velocity gai	Single - Har	Arithmetic	0.5-1	-5.06	0.95	126.57	-5.33	< .001
Velocity gai	Easy - Hard	Arithmetic	0.5-1	-0.2	0.75	372.08	-0.26	1
Velocity gai	Single - Eas	Arithmetic	1-1.5	-2.77	0.81	210.88	-3.41	0.002

For Review Only

Velocity gai	Single - Har	Arithmetic	1-1.5	-2.23	0.95	125.55	-2.36	0.06
Velocity gai	Easy - Hard	Arithmetic	1-1.5	0.54	0.74	367.16	0.72	1
Velocity gai	Single - Eas	Arithmetic	1.5-2	-1.98	0.81	208.35	-2.44	0.047
Velocity gai	Single - Har	Arithmetic	1.5-2	-1.49	0.95	125.15	-1.57	0.354
Velocity gai	Easy - Hard	Arithmetic	1.5-2	0.49	0.74	360.92	0.66	1
Velocity gai	Single - Eas	Arithmetic	2-2.5	-1.45	0.81	207.98	-1.79	0.225
Velocity gai	Single - Har	Arithmetic	2-2.5	-2	0.95	125.26	-2.11	0.11
Velocity gai	Easy - Hard	Arithmetic	2-2.5	-0.55	0.74	357.46	-0.74	1
Velocity gai	Single - Eas	Arithmetic	2.5-3	-2.36	0.81	206.62	-2.92	0.012
Velocity gai	Single - Har	Arithmetic	2.5-3	-2.86	0.95	124.78	-3.03	0.009
Velocity gai	Easy - Hard	Arithmetic	2.5-3	-0.5	0.74	353.2	-0.68	1
Velocity gai	Single - Eas	Arithmetic	3-3.5	-2.09	0.82	214.33	-2.56	0.033
Velocity gai	Single - Har	Arithmetic	3-3.5	-2.04	0.95	126.85	-2.15	0.1
Velocity gai	Easy - Hard	Arithmetic	3-3.5	0.05	0.74	367.46	0.06	1
Catch-up sa	Single - Eas	Arithmetic	0-0.5	0.05	0.03	665.71	1.86	0.192
Catch-up sa	Single - Har	Arithmetic	0-0.5	0.01	0.03	374.64	0.25	1
Catch-up sa	Easy - Hard	Arithmetic	0-0.5	-0.04	0.03	420.68	-1.52	0.389
Catch-up sa	Single - Eas	Arithmetic	0.5-1	0.11	0.03	721.98	4.21	< .001
Catch-up sa	Single - Har	Arithmetic	0.5-1	0.08	0.03	397.35	2.73	0.02
Catch-up sa	Easy - Hard	Arithmetic	0.5-1	-0.04	0.03	467.85	-1.27	0.61
Catch-up sa	Single - Eas	Arithmetic	1-1.5	0.04	0.03	705.66	1.36	0.527
Catch-up sa	Single - Har	Arithmetic	1-1.5	0.07	0.03	392.85	2.38	0.053
Catch-up sa	Easy - Hard	Arithmetic	1-1.5	0.03	0.03	459.81	1.09	0.831
Catch-up sa	Single - Eas	Arithmetic	1.5-2	0.03	0.03	689.12	1.21	0.685
Catch-up sa	Single - Har	Arithmetic	1.5-2	0	0.03	389.01	0.17	1
Catch-up sa	Easy - Hard	Arithmetic	1.5-2	-0.03	0.03	448.67	-0.97	0.995
Catch-up sa	Single - Eas	Arithmetic	2-2.5	0	0.03	688.6	0	1
Catch-up sa	Single - Har	Arithmetic	2-2.5	-0.05	0.03	389.19	-1.77	0.23
Catch-up sa	Easy - Hard	Arithmetic	2-2.5	-0.05	0.03	443.94	-1.78	0.227
Catch-up sa	Single - Eas	Arithmetic	2.5-3	0.03	0.03	683.05	0.94	1
Catch-up sa	Single - Har	Arithmetic	2.5-3	0.01	0.03	386.48	0.26	1
Catch-up sa	Easy - Hard	Arithmetic	2.5-3	-0.02	0.03	439.19	-0.63	1
Catch-up sa	Single - Eas	Arithmetic	3-3.5	0.06	0.03	726.65	2.26	0.073
Catch-up sa	Single - Har	Arithmetic	3-3.5	-0.02	0.03	400.81	-0.56	1
Catch-up sa	Easy - Hard	Arithmetic	3-3.5	-0.08	0.03	461.64	-2.71	0.021
Anticipator	Single - Eas	Arithmetic	0-0.5	-0.01	0.03	524.93	-0.31	1
Anticipator	Single - Har	Arithmetic	0-0.5	-0.02	0.03	391.9	-0.88	1
Anticipator	Easy - Hard	Arithmetic	0-0.5	-0.02	0.03	407.33	-0.58	1
Anticipator	Single - Eas	Arithmetic	0.5-1	0.1	0.03	570.03	3.83	< .001
Anticipator	Single - Har	Arithmetic	0.5-1	0.11	0.03	416.51	3.78	< .001
Anticipator	Easy - Hard	Arithmetic	0.5-1	0	0.03	454.66	0.07	1
Anticipator	Single - Eas	Arithmetic	1-1.5	0.02	0.03	556.62	0.92	1
Anticipator	Single - Har	Arithmetic	1-1.5	0.02	0.03	411.54	0.69	1
Anticipator	Easy - Hard	Arithmetic	1-1.5	-0.01	0.03	446.69	-0.21	1
Anticipator	Single - Eas	Arithmetic	1.5-2	-0.02	0.03	543.6	-0.62	1
Anticipator	Single - Har	Arithmetic	1.5-2	-0.01	0.03	407.38	-0.23	1
Anticipator	Easy - Hard	Arithmetic	1.5-2	0.01	0.03	435.51	0.37	1
Anticipator	Single - Eas	Arithmetic	2-2.5	0.01	0.03	543.13	0.49	1
Anticipator	Single - Har	Arithmetic	2-2.5	0.01	0.03	407.57	0.4	1
Anticipator	Easy - Hard	Arithmetic	2-2.5	0	0.03	430.79	-0.08	1

For Review Only

Anticipator	Single - Eas	Arithmetic	2.5-3	0.03	0.03	538.75	0.97	0.99
Anticipator	Single - Har	Arithmetic	2.5-3	0.05	0.03	404.66	1.63	0.313
Anticipator	Easy - Hard	Arithmetic	2.5-3	0.02	0.03	425.96	0.69	1
Anticipator	Single - Eas	Arithmetic	3-3.5	0	0.03	571.86	-0.18	1
Anticipator	Single - Har	Arithmetic	3-3.5	0.01	0.03	419.6	0.27	1
Anticipator	Easy - Hard	Arithmetic	3-3.5	0.01	0.03	447.68	0.44	1

For Review Only

Lower.CI	Upper.CI	Effect.Size	BF10	BF01
-0.06	0.09	0.02	0.2	5.07
-0.07	0.11	0.02	0.18	5.6
-0.08	0.08	0	0.15	6.5
-0.05	0.1	0.02	0.27	3.77
-0.06	0.12	0.03	0.27	3.71
-0.07	0.09	0.01	0.17	6.05
-0.05	0.1	0.03	0.35	2.82
-0.07	0.11	0.02	0.2	4.97
-0.08	0.07	-0.01	0.16	6.28
-0.06	0.09	0.02	0.2	5.1
-0.1	0.08	-0.01	0.17	5.94
-0.11	0.05	-0.03	0.31	3.27
-0.05	0.1	0.03	0.26	3.79
-0.12	0.06	-0.03	0.22	4.56
-0.13	0.03	-0.05	1.22	0.82
-0.05	0.1	0.02	0.25	4.06
-0.11	0.07	-0.02	0.18	5.47
-0.12	0.03	-0.04	0.59	1.69
-0.06	0.09	0.01	0.17	5.73
-0.1	0.08	-0.01	0.16	6.25
-0.1	0.06	-0.02	0.22	4.51
0.02	0.19	0.14	39.89	0.03
-0.03	0.15	0.08	1.15	0.87
-0.12	0.03	-0.06	0.91	1.1
0.08	0.25	0.22	3203.71	< .01
0.02	0.2	0.14	14.96	0.07
-0.13	0.02	-0.08	0.43	2.33
0.08	0.25	0.22	536.77	< .01
-0.01	0.18	0.11	1.96	0.51
-0.16	-0.01	-0.11	3.11	0.32
0.04	0.21	0.16	52.47	0.02
-0.04	0.14	0.06	0.36	2.81
-0.15	0	-0.1	2.09	0.48
0	0.17	0.12	4.87	0.21
-0.04	0.14	0.06	0.29	3.5
-0.12	0.03	-0.06	0.73	1.38
-0.01	0.16	0.1	2.06	0.49
-0.06	0.12	0.04	0.21	4.79
-0.12	0.03	-0.06	0.58	1.73
0	0.17	0.11	2.49	0.4
-0.05	0.13	0.05	0.27	3.65
-0.12	0.03	-0.06	0.54	1.85
-4.62	-0.72	-0.12	454.76	< .01
-4.89	-0.31	-0.12	29.85	0.03
-1.68	1.82	0	0.17	5.76
-6.84	-2.89	-0.23	50395.79	< .01
-7.37	-2.76	-0.24	> 100,000	< .01
-1.99	1.6	-0.01	0.16	6.19
-4.73	-0.81	-0.13	47.32	0.02

For Review Only

-4.53	0.07	-0.1	1.54	0.65
-1.25	2.32	0.03	0.23	4.27
-3.94	-0.02	-0.09	21.1	0.05
-3.79	0.81	-0.07	0.72	1.39
-1.29	2.27	0.02	0.18	5.44
-3.41	0.51	-0.07	0.95	1.06
-4.3	0.3	-0.09	2.35	0.43
-2.32	1.23	-0.03	0.32	3.15
-4.32	-0.41	-0.11	30.06	0.03
-5.16	-0.57	-0.13	9.33	0.11
-2.27	1.27	-0.02	0.22	4.49
-4.06	-0.12	-0.1	11.9	0.08
-4.35	0.26	-0.1	1.92	0.52
-1.74	1.83	0	0.16	6.31
-0.01	0.11	0.06	1.27	0.79
-0.06	0.07	0.01	0.18	5.53
-0.11	0.02	-0.05	0.51	1.98
0.05	0.18	0.13	20.92	0.05
0.01	0.15	0.09	2.21	0.45
-0.11	0.03	-0.04	0.29	3.42
-0.03	0.1	0.04	0.25	4.02
0	0.14	0.08	1.65	0.6
-0.04	0.1	0.04	0.29	3.49
-0.03	0.1	0.04	0.41	2.45
-0.06	0.07	0.01	0.19	5.3
-0.1	0.04	-0.03	0.2	4.96
-0.06	0.06	0	0.16	6.37
-0.12	0.02	-0.06	0.57	1.76
-0.12	0.02	-0.06	0.85	1.18
-0.04	0.09	0.03	0.26	3.8
-0.06	0.08	0.01	0.16	6.23
-0.09	0.05	-0.02	0.2	5.09
0	0.13	0.07	2.62	0.38
-0.08	0.05	-0.02	0.18	5.49
-0.15	-0.01	-0.09	9.5	0.11
-0.07	0.06	-0.01	0.16	6.21
-0.09	0.04	-0.03	0.26	3.91
-0.08	0.05	-0.02	0.23	4.33
0.04	0.17	0.12	772.39	< .01
0.04	0.17	0.12	281.21	< .01
-0.07	0.07	0	0.16	6.34
-0.04	0.09	0.03	0.2	5.07
-0.05	0.09	0.02	0.18	5.56
-0.07	0.06	-0.01	0.16	6.37
-0.08	0.05	-0.02	0.19	5.37
-0.07	0.06	-0.01	0.16	6.37
-0.06	0.08	0.01	0.17	5.77
-0.05	0.08	0.02	0.17	5.88
-0.06	0.08	0.01	0.16	6.16
-0.07	0.06	0	0.16	6.35

For Review Only

-0.04	0.09	0.03	0.23	4.29
-0.02	0.11	0.05	0.66	1.51
-0.05	0.09	0.02	0.21	4.71
-0.07	0.06	-0.01	0.16	6.34
-0.06	0.07	0.01	0.16	6.09
-0.05	0.08	0.01	0.17	5.96

For Review Only

Contrast	Workload	Task	Estimate	*SE*	*df*	*t*	*p*
0-0.5 - 0.5-1	Single	Arithmetic	0.02	0.01	204478.4	1.97	0.293
0.5-1 - 1-1.5	Single	Arithmetic	0.01	0.01	204478.4	1.06	1
1-1.5 - 1.5-2	Single	Arithmetic	-0.01	0.01	204478.4	-1.18	1
1.5-2 - 2-2.5	Single	Arithmetic	-0.01	0.01	204478.5	-1.85	0.382
2-2.5 - 2.5-3	Single	Arithmetic	-0.01	0.01	204478.5	-1.72	0.517
2.5-3 - 3-3.5	Single	Arithmetic	-0.02	0.01	204547.6	-2.52	0.071
0-0.5 - 0.5-1	Easy	Arithmetic	0.03	0.01	204478.5	3.98	< .001
0.5-1 - 1-1.5	Easy	Arithmetic	0.05	0.01	204478.5	6.63	< .001
1-1.5 - 1.5-2	Easy	Arithmetic	-0.01	0.01	204478.5	-1.58	0.69
1.5-2 - 2-2.5	Easy	Arithmetic	-0.06	0.01	204478.5	-7.3	< .001
2-2.5 - 2.5-3	Easy	Arithmetic	-0.03	0.01	204478.5	-4.47	< .001
2.5-3 - 3-3.5	Easy	Arithmetic	-0.02	0.01	204602	-2.14	0.192
0-0.5 - 0.5-1	Hard	Arithmetic	0.02	0.01	204478.3	3.07	0.013
0.5-1 - 1-1.5	Hard	Arithmetic	0.08	0.01	204478.4	10.07	< .001
1-1.5 - 1.5-2	Hard	Arithmetic	0.03	0.01	204478.7	3.38	0.004
1.5-2 - 2-2.5	Hard	Arithmetic	-0.03	0.01	204478.7	-3.84	< .001
2-2.5 - 2.5-3	Hard	Arithmetic	-0.05	0.01	204478.9	-6.19	< .001
2.5-3 - 3-3.5	Hard	Arithmetic	-0.04	0.01	204547.7	-5.35	< .001
0-0.5 - 0.5-1	Single	Visuospatia	0.01	0.01	204478.4	1.45	0.878
0.5-1 - 1-1.5	Single	Visuospatia	0	0.01	204478.4	0.12	1
1-1.5 - 1.5-2	Single	Visuospatia	0	0.01	204478.3	-0.5	1
1.5-2 - 2-2.5	Single	Visuospatia	-0.01	0.01	204478.5	-1.93	0.322
2-2.5 - 2.5-3	Single	Visuospatia	-0.01	0.01	204478.5	-1.13	1
2.5-3 - 3-3.5	Single	Visuospatia	-0.01	0.01	204513.6	-1.84	0.394
0-0.5 - 0.5-1	Easy	Visuospatia	0.03	0.01	204478.4	4.33	< .001
0.5-1 - 1-1.5	Easy	Visuospatia	0.05	0.01	204478.5	6.33	< .001
1-1.5 - 1.5-2	Easy	Visuospatia	-0.02	0.01	204478.7	-2.39	0.101
1.5-2 - 2-2.5	Easy	Visuospatia	-0.05	0.01	204478.6	-6.21	< .001
2-2.5 - 2.5-3	Easy	Visuospatia	-0.03	0.01	204478.5	-4.04	< .001
2.5-3 - 3-3.5	Easy	Visuospatia	-0.02	0.01	204567.2	-3.04	0.014
0-0.5 - 0.5-1	Hard	Visuospatia	0.03	0.01	204478.5	4.48	< .001
0.5-1 - 1-1.5	Hard	Visuospatia	0.06	0.01	204478.6	7.99	< .001
1-1.5 - 1.5-2	Hard	Visuospatia	0	0.01	204478.6	-0.45	1
1.5-2 - 2-2.5	Hard	Visuospatia	-0.04	0.01	204478.5	-5.69	< .001
2-2.5 - 2.5-3	Hard	Visuospatia	-0.04	0.01	204478.4	-4.67	< .001
2.5-3 - 3-3.5	Hard	Visuospatia	-0.03	0.01	204513.3	-3.59	0.002

For Review Only

Lower.CL	Upper.CL	Effect.Size	BF10	BF01
-0.01	0.04	0.02	16435.92	< 0.01
-0.01	0.03	0.01	23.59	0.04
-0.03	0.01	-0.01	19.44	0.05
-0.03	0.01	-0.01	7673.08	< 0.01
-0.03	0.01	-0.01	90703.6	< 0.01
-0.04	0	-0.02	> 100,000	< 0.01
0.01	0.05	0.03	> 100,000	< 0.01
0.03	0.07	0.05	> 100,000	< 0.01
-0.03	0.01	-0.01	4.11	0.24
-0.08	-0.04	-0.06	> 100,000	< 0.01
-0.05	-0.01	-0.04	> 100,000	< 0.01
-0.04	0	-0.02	21.22	0.05
0	0.04	0.02	7769.25	< 0.01
0.06	0.1	0.08	> 100,000	< 0.01
0.01	0.05	0.03	> 100,000	< 0.01
-0.05	-0.01	-0.03	> 100,000	< 0.01
-0.07	-0.03	-0.05	> 100,000	< 0.01
-0.06	-0.02	-0.04	> 100,000	< 0.01
-0.01	0.03	0.01	64.5	0.02
-0.02	0.02	0	0.16	6.21
-0.02	0.02	0	0.51	1.95
-0.04	0.01	-0.02	5309.79	< 0.01
-0.03	0.01	-0.01	9.06	0.11
-0.03	0.01	-0.01	4934.07	< 0.01
0.01	0.05	0.03	> 100,000	< 0.01
0.03	0.07	0.05	> 100,000	< 0.01
-0.04	0	-0.02	36.27	0.03
-0.07	-0.03	-0.05	> 100,000	< 0.01
-0.05	-0.01	-0.03	> 100,000	< 0.01
-0.04	0	-0.02	2093.14	< 0.01
0.01	0.05	0.04	> 100,000	< 0.01
0.04	0.08	0.06	> 100,000	< 0.01
-0.02	0.02	0	0.2	4.94
-0.06	-0.02	-0.04	> 100,000	< 0.01
-0.06	-0.02	-0.04	> 100,000	< 0.01
-0.05	-0.01	-0.03	> 100,000	< 0.01

For Review Only

Eye.Parame	Effect	*Sum.Sq*	*Mean.Sq*	*Num.DF*	*Den.DF*	*F*	*p*
Catch-up sa	Task	0.17	0.17	1	48.83	0.26	.609
Catch-up sa	Load	31.26	15.63	2	57.85	24.32	< .01
Catch-up sa	Time	208.19	34.7	6	185017.41	53.99	< .01
Catch-up sa	(Task) orde	0.01	0.01	1	44.93	0.02	.883
Catch-up sa	Task:load	6.05	3.03	2	64.5	4.71	.012
Catch-up sa	Task:time	14.24	2.37	6	185024.97	3.69	< .01
Catch-up sa	Load:time	101.92	8.49	12	185009.99	13.22	< .01
Catch-up sa	Task:load:ti	19.58	1.63	12	185013.11	2.54	< .01
Anticipator	Task	1.95	1.95	1	48.27	3.07	.086
Anticipator	Load	10.67	5.34	2	53.94	8.4	< .01
Anticipator	Time	37.35	6.22	6	185052.3	9.79	< .01
Anticipator	(Task) orde	0.65	0.65	1	45.77	1.02	.318
Anticipator	Task:load	1.50	0.75	2	56.33	1.18	.315
Anticipator	Task:time	15.90	2.65	6	185057.06	4.17	< .01
Anticipator	Load:time	26.66	2.22	12	185043.67	3.49	< .01
Anticipator	Task:load:ti	15.98	1.33	12	185046.65	2.1	.014

For Review Only

Appendix B: Supplementary Figures 1-3

Figure B1

Velocity gain based on angular velocity during SPEM for task modality (arithmetic vs. visuospatial) across the trial duration for all workload conditions (single task, low and high dual task)

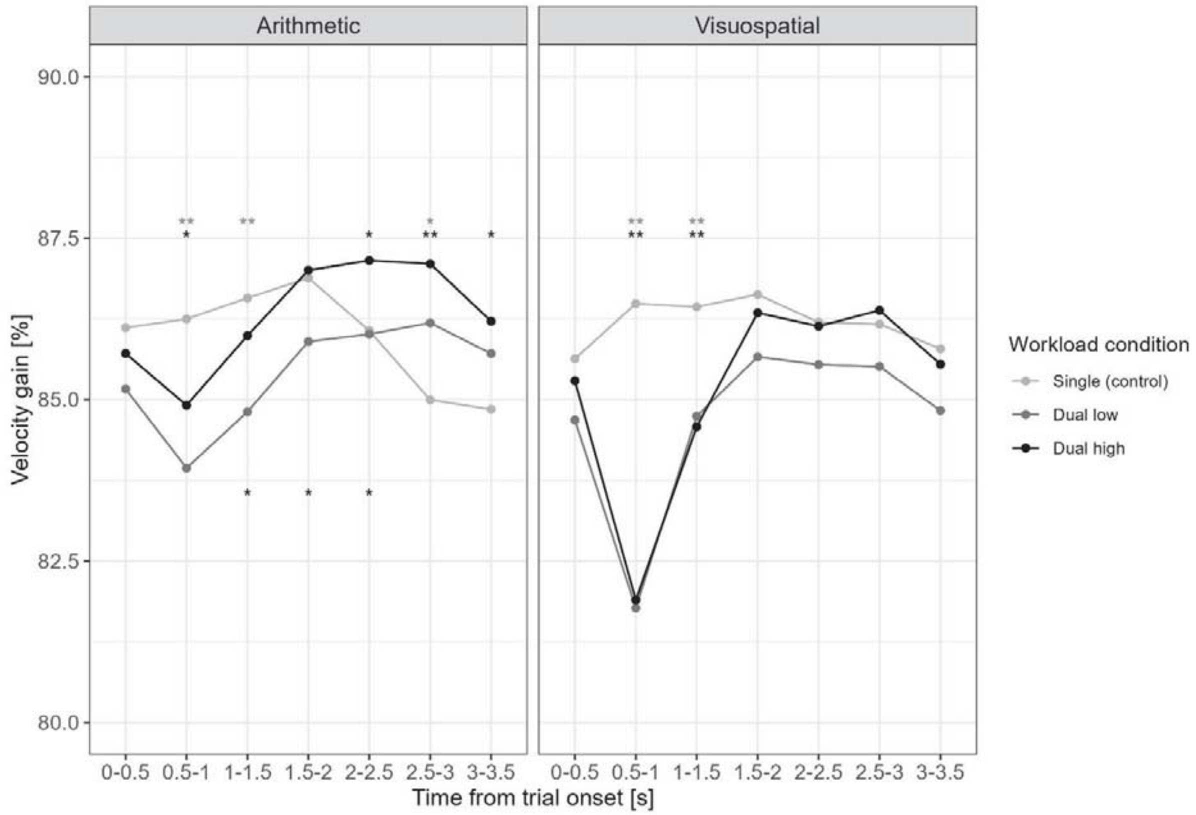

Note. Target position data was up sampled from 30 to 1000 Hz and then together with gaze position transformed from degrees of visual angle to angular degrees. Gaze angular position data was smoothed with a rolling mean over 100 samples and samples around the "end" of a circle, i.e., when gaze position moves from 360° to 0°, were excluded. Velocity gain was calculated as a ratio between gaze's and target's angular position. However, calculation of gaze's angular velocity led to an increase of missing values, which ultimately resulted in the exclusion of 13 datasets (7 of which from the arithmetic task). These results should thus be interpreted and compared to the main results with caution.

Statistically significant differences are presented with asterisks (* $p < .05$; ** $p < .01$). Asterisks near the single condition line represent significant differences between single vs. low condition

(top row, grey) and single vs. high condition (bottom row, black). Asterisks near the high condition line represent significant differences between low vs. high condition (black).

Figure B2

Catch-up-saccades (C) and anticipatory saccades (D) based on angular degrees during SPEM for task modality (arithmetic vs. visuospatial) across the trial duration for all workload conditions (single task, low and high dual task)

For Review Only

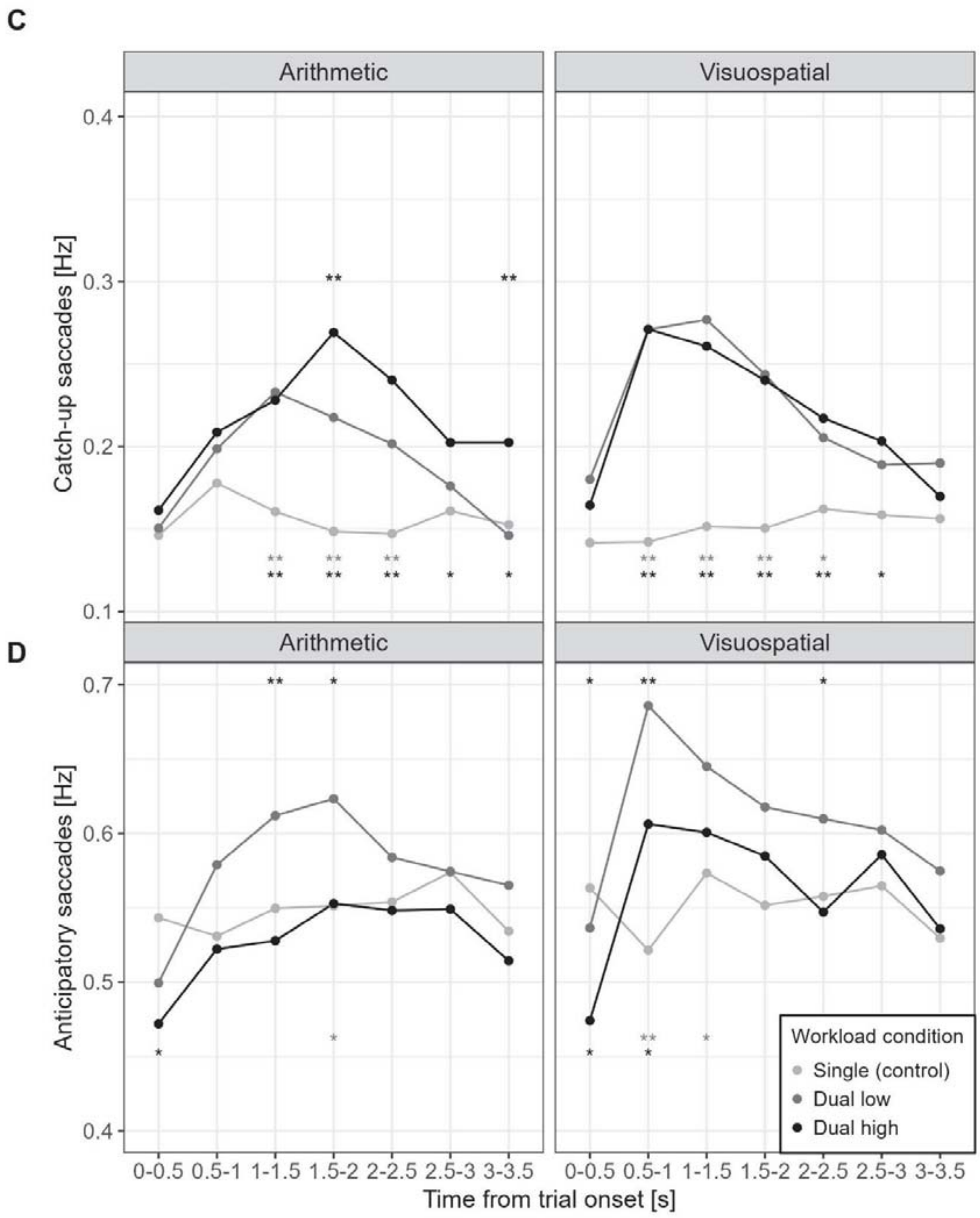

Note. Catch-up and anticipatory saccades were defined based on gaze's angular position. Saccades that landed behind the target were labeled as catch-up, and those landing in front of the target as anticipatory. Statistically significant differences are presented with asterisks (* p < .05; ** p < .01). Asterisks in the bottom represent significant difference between single vs. low condition (first row, grey) and single vs. high condition (bottom row, black). Asterisks on the top present significant difference between low versus high condition.

Figure B3

Anticipatory saccades during SPEM for the visuospatial task in the second time-bin for all workload conditions (single task, dual tasks)

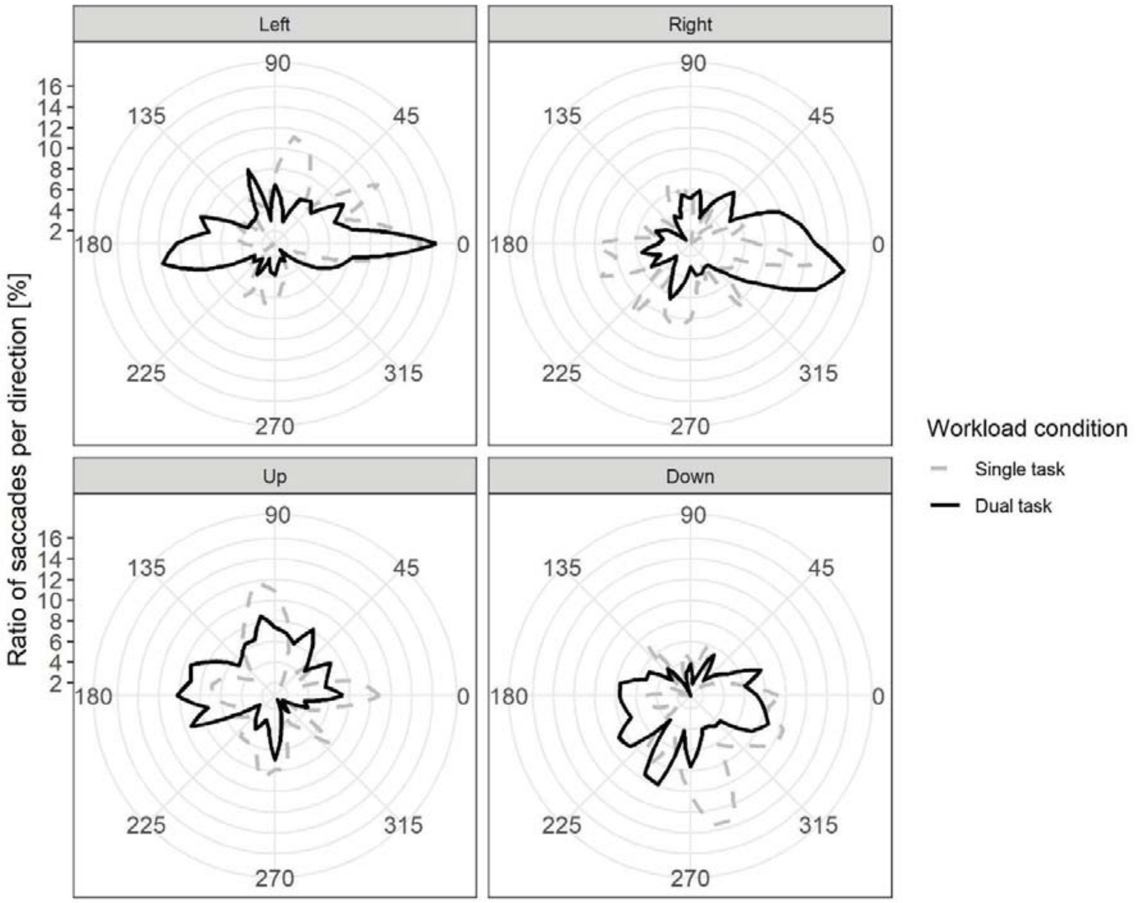

Note. Illustration of the proportions of anticipatory saccades directions from time-bin 2, pooled low and high dual conditions in the visuospatial task. Saccades are depicted in four panels corresponding to the audio instruction for the internal operation (left, right, up, down).
